# Gut microbial community and fecal metabolomic signatures in different types of osteoporosis animal models

**DOI:** 10.18632/aging.205396

**Published:** 2024-01-26

**Authors:** Xiaochen Qiao, Xiaoyan Li, Zhichao Wang, Yi Feng, Xiaochun Wei, Lu Li, Yongchun Pan, Kun Zhang, Ruhao Zhou, Lei Yan, Pengcui Li, Chaojian Xu, Zhi Lv, Zhi Tian

**Affiliations:** 1 Second Clinical Medical College, Shanxi Medical University, Taiyuan 030001, Shanxi, P.R. China; 2 Department of Orthopedics, The Second Hospital of Shanxi Medical University, Shanxi Key laboratory of Bone and Soft Tissue Injury Repair, Taiyuan 030001, Shanxi, P.R. China; 3 Department of Orthopedics, Jinzhong Hospital Affiliated to Shanxi Medical University, Jinzhong 030600, Shanxi, P.R. China; 4 Shanxi Province Cancer Hospital, Shanxi Hospital Affiliated to Cancer Hospital, Chinese Academy of Medical Sciences, Cancer Hospital Affiliated to Shanxi Medical University, Taiyuan 030013, Shanxi, P.R. China; 5 Third Hospital of Shanxi Medical University, Shanxi Bethune Hospital, Shanxi Academy of Medical Sciences, Tongji Shanxi Hospital, Taiyuan 030032, Shanxi, China; 6 Department of Orthopedics, Third People’s Hospital of Datong City, Datong 037006, Shanxi, P.R. China

**Keywords:** osteoporosis, animal model, gut microbiota, 16S rDNA sequencing, metabolomics

## Abstract

Background: The gut microbiota (GM) constitutes a critical factor in the maintenance of physiological homeostasis. Numerous studies have empirically demonstrated that the GM is closely associated with the onset and progression of osteoporosis (OP). Nevertheless, the characteristics of the GM and its metabolites related to different forms of OP are poorly understood. In the present study, we examined the changes in the GM and its metabolites associated with various types of OP as well as the correlations among them.

Methods: We simultaneously established rat postmenopausal, disuse-induced, and glucocorticoid-induced OP models. We used micro-CT and histological analyses to observe bone microstructure, three-point bending tests to measure bone strength, and enzyme-linked immunosorbent assay (ELISA) to evaluate the biochemical markers of bone turnover in the three rat OP models and the control. We applied 16s rDNA to analyze GM abundance and employed untargeted metabolomics to identify fecal metabolites in all four treatment groups. We implemented multi-omics methods to explore the relationships among OP, the GM, and its metabolites.

Results: The 16S rDNA sequencing revealed that both the abundance and alterations of the GM significantly differed among the OP groups. In the postmenopausal OP model, the bacterial genera g__Bacteroidetes_unclassified, g__Firmicutes_unclassified, and g__Eggerthella had changed. In the disuse-induced and glucocorticoid-induced OP models, g__Akkermansia and g__Rothia changed, respectively. Untargeted metabolomics disclosed that the GM-derived metabolites significantly differed among the OP types. However, a Kyoto Encyclopedia of Genes and Genomes (KEGG) enrichment analysis showed that it was mainly metabolites implicated in lipid and amino acid metabolism that were altered in all cases. An association analysis indicated that the histidine metabolism intermediate 4-(β-acetylaminoethyl) imidazole was common to all OP forms and was strongly correlated with all bone metabolism-related bacterial genera. Hence, 4-(β-acetylaminoethyl) imidazole might play a vital role in OP onset and progression.

Conclusions: The present work revealed the alterations in the GM and its metabolites that are associated with OP. It also disclosed the changes in the GM that are characteristic of each type of OP. Future research should endeavor to determine the causal and regulatory effects of the GM and the metabolites typical of each form of OP.

## INTRODUCTION

Osteoporosis (OP) is characterized by low bone mineral density, bone architecture deterioration, and increased risk of fracture, and has become a major global health problem [1]. Approximately 200 million people worldwide suffer from OP and nine million OP-related fractures occur annually [2]. Bone fracture is the main complication of OP and is associated with increased morbidity and mortality [3]. The incidence of OP is expected to continue to rise. The disease will diminish the quality of life of the aging global population and impose a huge socioeconomic burden on society at large [4, 5]. OP is classified as primary or secondary [6]. Postmenopausal, disuse, and glucocorticoid-induced OP are the major forms of the disorder in humans. Of these, postmenopausal OP is primary while the other two are secondary [7]. At present, the treatment of OP is still based on drug therapy [8]. Long-term anti-osteoporotic drug administration may cause mandibular osteonecrosis and atypical femoral fracture [9, 10]. Therefore, the development of novel therapeutic approaches against bone loss is a priority.

The gut microbiota (GM) comprises the commensal microorganisms that inhabit the human intestines and function as a secondary gene pool [11, 12]. The GM helps regulate various physiological functions and is associated with various diseases of muscle and bone metabolism [12–16]. Dynamic GM homeostasis is vital to health. When the GM is altered and this balance is perturbed, the host may develop certain pathological conditions. The GM may strongly influence bone metabolism, and GM modulation could reverse bone loss. Hence, the GM is a potential target of OP treatment [17, 18]. The GM strongly affects metabolism and the immune system in humans and animals [19]. The correlation between the GM and the immune system is crucial as the latter helps regulate bone density [20]. GM dysbiosis is closely associated with an increased risk of bone loss [17]. Hence, it is necessary to explore the relationship between bone health and the GM, study the role of the latter in osteoporosis, and apply it in the clinical treatment of this disorder [21].

The gut microbial community and the fecal metabolomic signatures related to postmenopausal, disuse, and glucocorticoid-induced osteoporosis remain unknown. Animal models are currently being established and implemented to explore gut-bone interaction as the gut metagenome has been characterized [22]. In the present study, we applied various techniques to construct animal models of ovariectomized (OVX), disuse-induced (DIO), and glucocorticoid-induced (GIO) OP. We then performed 16S rDNA gene sequencing and untargeted liquid chromatography-mass spectrometry (LC-MS)-based metabolomics on feces to explore the GM and the modifications to metabolites in various OP models. Understanding the GM and its metabolites characteristic of each type of OP could facilitate the development and administration of novel therapeutic approaches against this condition.

## MATERIALS AND METHODS

### Animals

Female Sprague-Dawley (SD) rats aged 12 weeks were obtained from the Ying Ze District Campus Animal Testing Center of Shanxi Medical University, Shanxi, China. They were housed under specific-pathogen-free (SPF) conditions and a 12 h light/12 h dark cycle, and had *ad libitum* access to sterile food and autoclaved water. They were subjected to 1 week of adaptive feeding and randomly divided into four groups of six rats per group. The treatments included (1) bilateral ovariectomy-induced postmenopausal OP (OVX) [23], (2) right leg sciatic neurotomy-induced disuse OP (DIO) [24], (3) glucocorticoid-induced OP established by 1 mg kg^–1^ intramuscular dexamethasone saline injection every other day (GIO) [25], and (4) an untreated control (CON). After 10 weeks, rats were rendered unconscious by CO2 inhalation and sacrificed. Blood and femurs of the rats were collected for further analysis.

### Micro-CT

Micro-CT (vivaCT80; SCANCO Medical AG, Wangen-Brüttisellen, Switzerland) was used to scan the distal femurs and compare the trabecular bones among the models. The parameters evaluated were bone mineral density (BMD), bone volume per tissue volume (BV/TV), trabecular spacing (Tb.Sp), trabecular number (Tb.N), trabecular thickness (Tb.Th), and the structure model index (SMI).

### Histological analysis

Femoral samples were excised, fixed in 4% (v/v) paraformaldehyde (PFA), decalcified in 20% (w/v) ethylenediaminetetraacetic acid (EDTA), and cut into 5-mm sagittal sections that were stained with hematoxylin and eosin (H&E) and examined under a light microscope.

### Mechanical tests

A three-point bending test (ElectroForce 3200 Series, TA Instruments, New Castle, DE, USA) was performed to measure mechanical stress on the femurs. The metrics evaluated included maximum displacement, fracture load, peak load, and stiffness.

### ELISA

Blood was drawn from the abdominal aorta and centrifuged at 3,000 rpm for 15 min to obtain the serum. ELISA kits (Lunchang Shuo Biotechnology, Xiamen, China) were used to measure the serum N-terminal propeptide of type I procollagen (PINP) and C-terminal telopeptide of type I collagen (CTX-I) levels.

### Fecal sampling

After 10 weeks of animal maintenance, sufficient fecal samples were collected from the rats and subjected to microbial and metabolic analyses. All fecal samples were placed in sterile centrifuge tubes, immediately frozen in liquid nitrogen, and stored at -80° C until sequencing.

### 16S rDNA sequencing and microbial community analysis

The 16S rDNA sequencing was conducted at Lc-Bio Technologies Co. Ltd., Hangzhou, Zhejiang, China. The cetyltrimethylammonium ammonium bromide (CTBA) method was used to extract total DNA from all samples. Polymerase chain reaction (PCR) amplification of the V3–V4 region of the 16S rRNA gene was performed using the 341F (5`-CCTACGGGNGGCWGCAG-3`) and 805R (5`-GACTACHVGGGTATCTAATCC-3`) primers [26]. The PCR was performed as follows: initial denaturation at 98° C for 30 s, 32 denaturation cycles at 98° C for 10 s, annealing at 54° C for 30 s, extension at 72° C for 45 s, and final extension at 72° C for 10 min. The PCR product size was confirmed by 2% agarose gel electrophoresis. AMPure XT beads (Beckman Coulter Genomics, Danvers, MA, USA) and Qubit (Invitrogen, Carlsbad, CA, USA) were applied to purify and quantify the PCR products. Next, the amplicon pools were applied for sequencing and the libraries were sequenced on the NovaSeq 6000 platform (Illumina, San Diego, CA, USA). After quality filtering, high quality clean tags were obtained using FQTRIM (v.0.94, http://ccb.jhu.edu/software/fqtrim/). Next, amplicon sequence variant (ASV) feature tables and sequences were obtained using DADA2 (v2019.7, https://qiime2.org/) to denoise. Finally, diversity Analysis, species annotation, difference analysis, and advanced analysis were performed based on ASV feature tables and sequences obtained above.

### Untargeted metabolomics data analysis

Fifty milligrams of each frozen sample were set aside, transferred to a 1.5-mL Eppendorf (EP) tube (Eppendorf GmbH, Hamburg, Germany), and thawed on ice. The metabolites were extracted with 50% (v/v) methanol buffer-acetonitrile and centrifuged at 4,000 × *g* for 20 min. The supernatants were stored at -80° C until they were subjected to liquid chromatography-mass spectrometry (LC-MS). The LC-MS was performed in a Thermo Scientific UltiMate 3000 HPLC system (Thermo Fisher Scientific, Waltham, MA, USA) coupled to a high-resolution tandem Q-Exactive MS (Thermo Fisher Scientific, Waltham, MA, USA) operated in positive and negative ion modes [26]. An online Kyoto Encyclopedia of Genes and Genomes (KEGG) database (https://www.genome.jp/kegg/) annotated the metabolites by matching their exact molecular mass data, names, and formulae with those in the database. A principal component analysis (PCA) was performed to detect outliers in the preprocessed dataset. Differential metabolites had variable influence of projection (VIP) > 1, P < 0.05, and ratio ≥ 2 or ≤ ½, and a KEGG enrichment analysis was performed on them [27–29]. Correlations between significant differential genera and metabolites were analyzed by Spearman’s rank correlation test.

### Statistical analysis

All data were presented as means ± standard deviation (SD). Differences between group pairs were analyzed by Student’s *t*-test. Multiple group comparisons were performed by one-way analysis of variance (ANOVA) with the Bonferroni correction. Wilcoxon’s rank-sum and Kruskal-Wallis tests were applied to identify differences in the bacterial taxa between group pairs and multiple groups, respectively. Student’s *t*-test and fold change (FC) analysis were used to identify differences in metabolites between groups.

### Availability of data and materials

The original data used in this study are publicly available at https://www.ncbi.nlm.nih.gov/sra/PRJNA973838.

## RESULTS

### Alterations in the femoral bone microarchitecture of various OP models in rats

**Figure 1:**
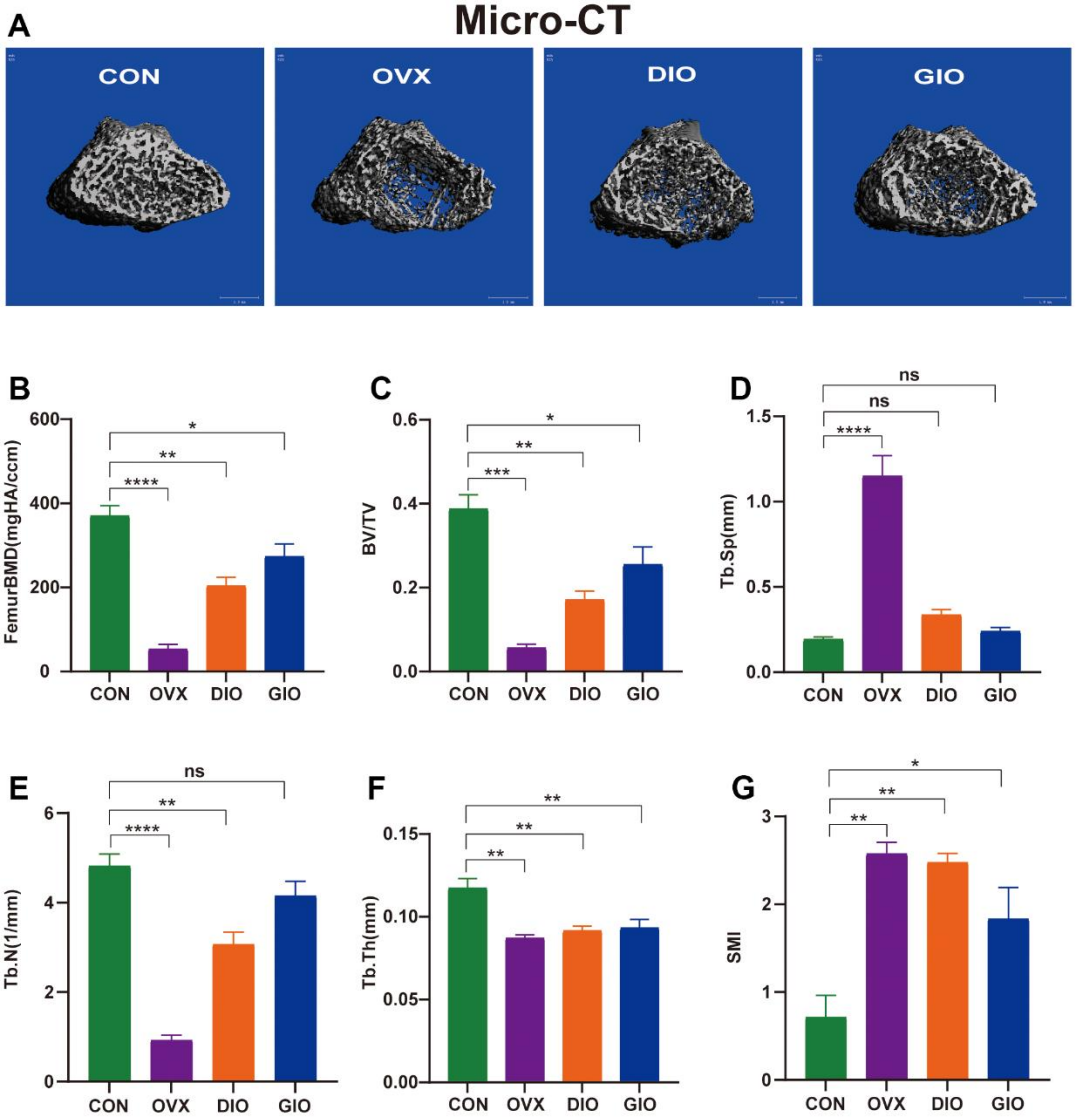
(**A**) Representative 3D micro-CT reconstructions of femurs from per group. (**B**–**G**) Trabecular bone at distal femoral metaphysis after 10 weeks. Parameters included BMD, BV/TV, Tb.Sp, Tb.N, Tb.Th, and SMI. Data are means ± standard error of the mean (SEM). n = 6, *P < 0.05, **P < 0.01, ***P < 0.001, and ****P < 0.0001; ns, no significance.

We performed micro-CT analyses in all groups. The 3D micro-CT images revealed obvious changes in the distal femoral metaphysis bone microstructure in the OVX, DIO, and GIO groups compared with the CON group (Figure 1A). The micro-CT disclosed that the femoral BMD, BV/TV, Tb.N, and Tb.Th values were lower for the OVX, DIO, and GIO groups than the CON group (Figure 1B–1F). In contrast, the Tb.Sp and SMI values were higher for the OVX, DIO, and GIO groups than the CON group (Figure 1D, 1G).

### Changes in the bone histomorphology, mechanical properties, and serum bone turnover markers of various OP models in rats

**Figure 2:**
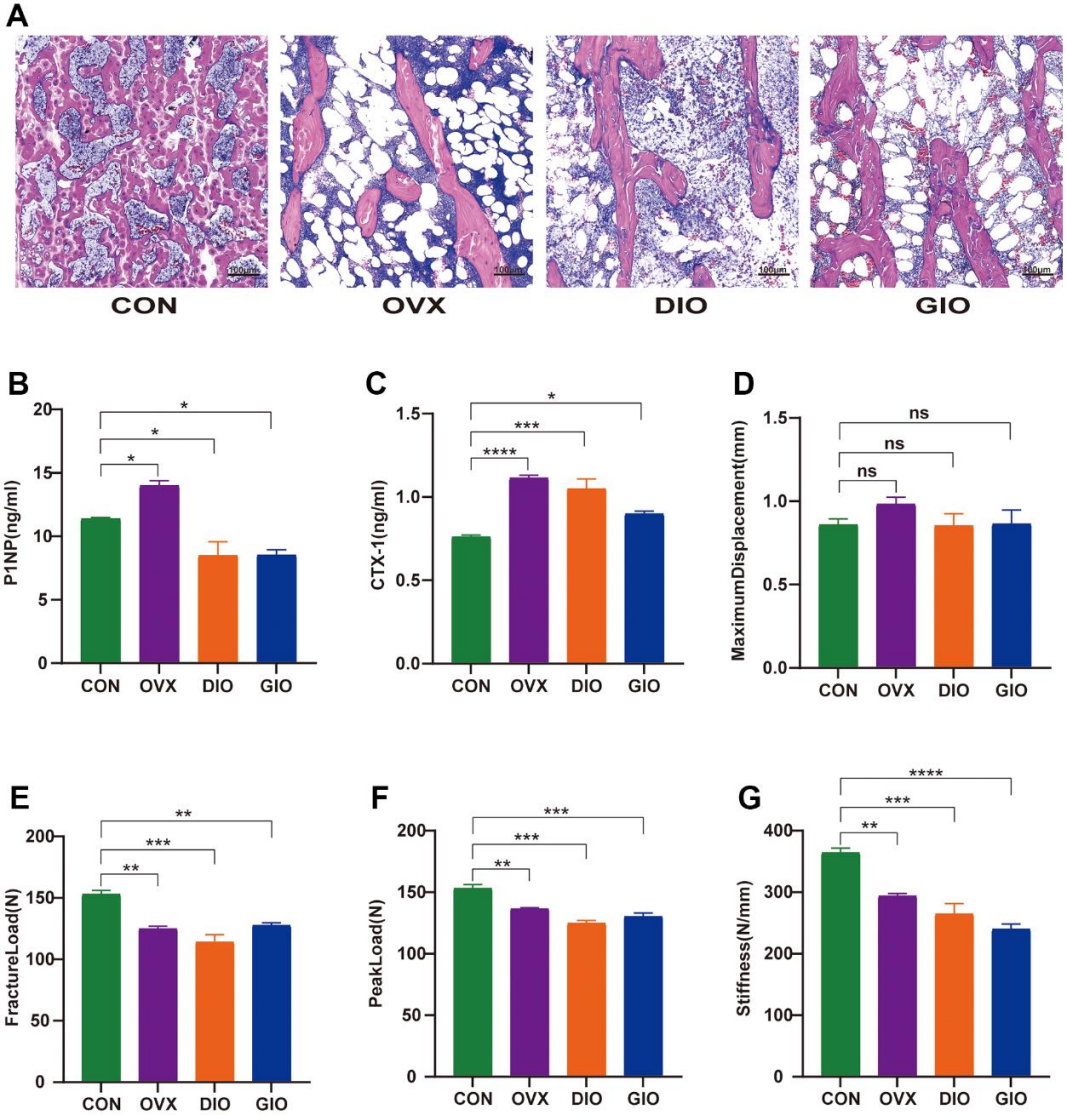
(**A**) Trabecular bone at distal femoral metaphysis observed by H&E staining. (**B**, **C**) Serum levels of bone turnover biomarkers including PINP and CTX-I. (**D**–**G**) Comparison of three-point bending test parameters including maximum displacement, fracture load, peak load, and stiffness. Data are means ± SEM. n = 6, *P < 0.05, **P < 0.01, ***P < 0.001, and ****P < 0.0001; ns, no significance.

Hematoxylin-eosin (H&E) staining, a three-point bending test, and an enzyme-linked immunosorbent assay (ELISA) were used to examine and compare the bone histomorphology, mechanical properties, and serum bone turnover indices. The H&E staining showed that compared with the CON group, the numbers of bone trabeculae were significantly reduced and the bone trabeculae were rod-shaped rather than plate-like in the OVX, DIO, and GIO groups. These findings were consistent with the SMI values determined by micro-CT. Moreover, the bone marrow adipocyte counts were higher in the OVX, DIO, and GIO groups than in the CON group (Figure 2A). The bone resorption and formation markers include CTX-I and PINP, respectively. The ELISA showed that the serum PINP was relatively lower in the DIO and GIO models and higher in the OVX model because high bone turnover is associated with postmenopausal OP. The serum CTX-I levels were higher in the OVX, DIO, and GIO groups than in the CON group (Figure 2B, 2C). The three-point bending test disclosed that the fracture load, peak load, and stiffness were significantly lower in the OVX, DIO, and GIO groups than in the CON group. However, there were no significant differences among treatments in terms of the maximum displacement (Figure 2D–2G).

### Relative differences in the gut microbiota among various OP models in rats

**Figure 3:**
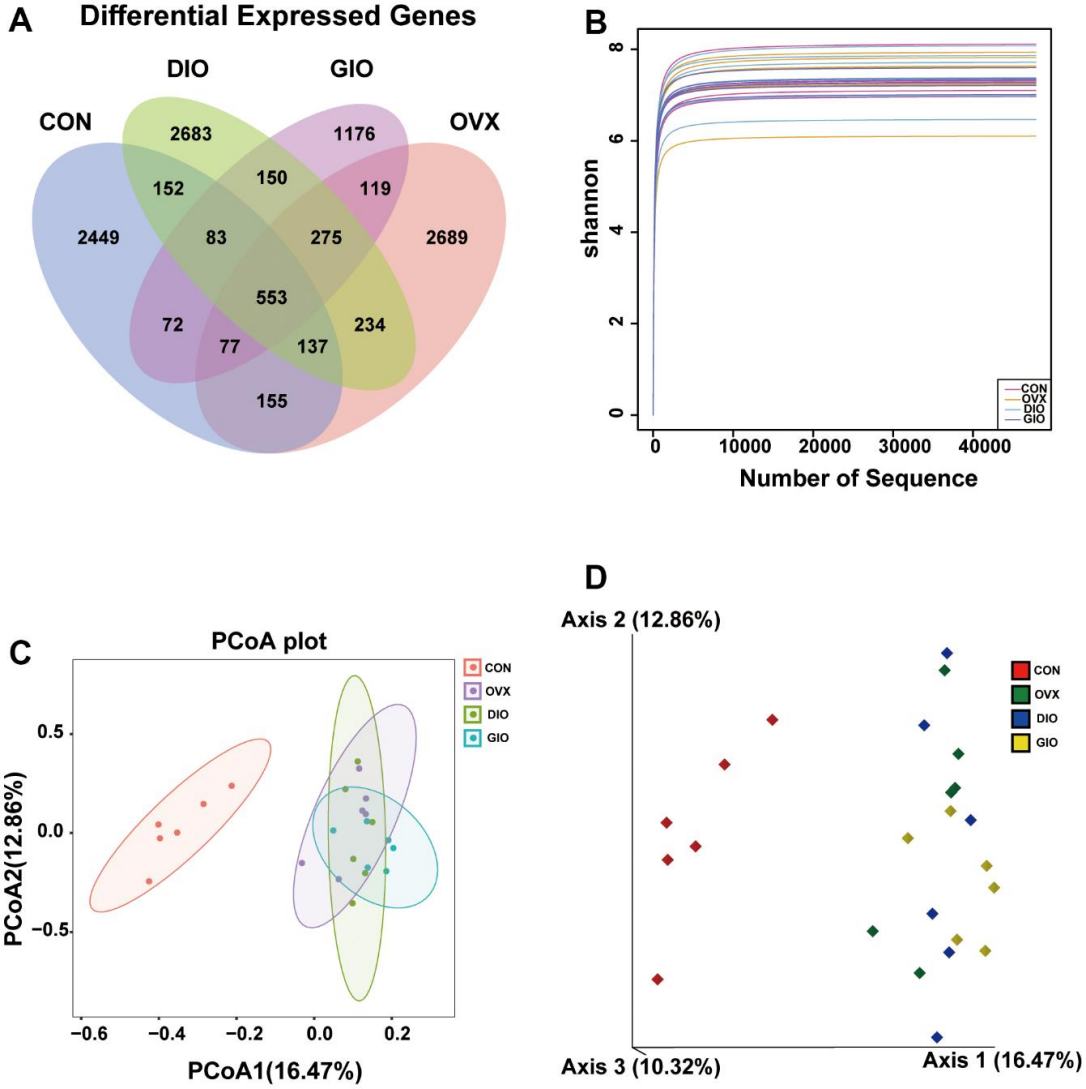
(**A**) Venn diagram showing numbers of amplicon sequence variants (ASVs) per group. (**B**) Rarefaction curves of Shannon index α-diversity analysis. (**C**) 2D model of gut microbiota PCoA. CON: orange; OVX: purple; DIO: light green; GIO: dark green. (**D**) 3D model of gut microbiota PCoA. CON: red; OVX: green; DIO: blue; GIO: yellow; n = 6.

**Figure 4:**
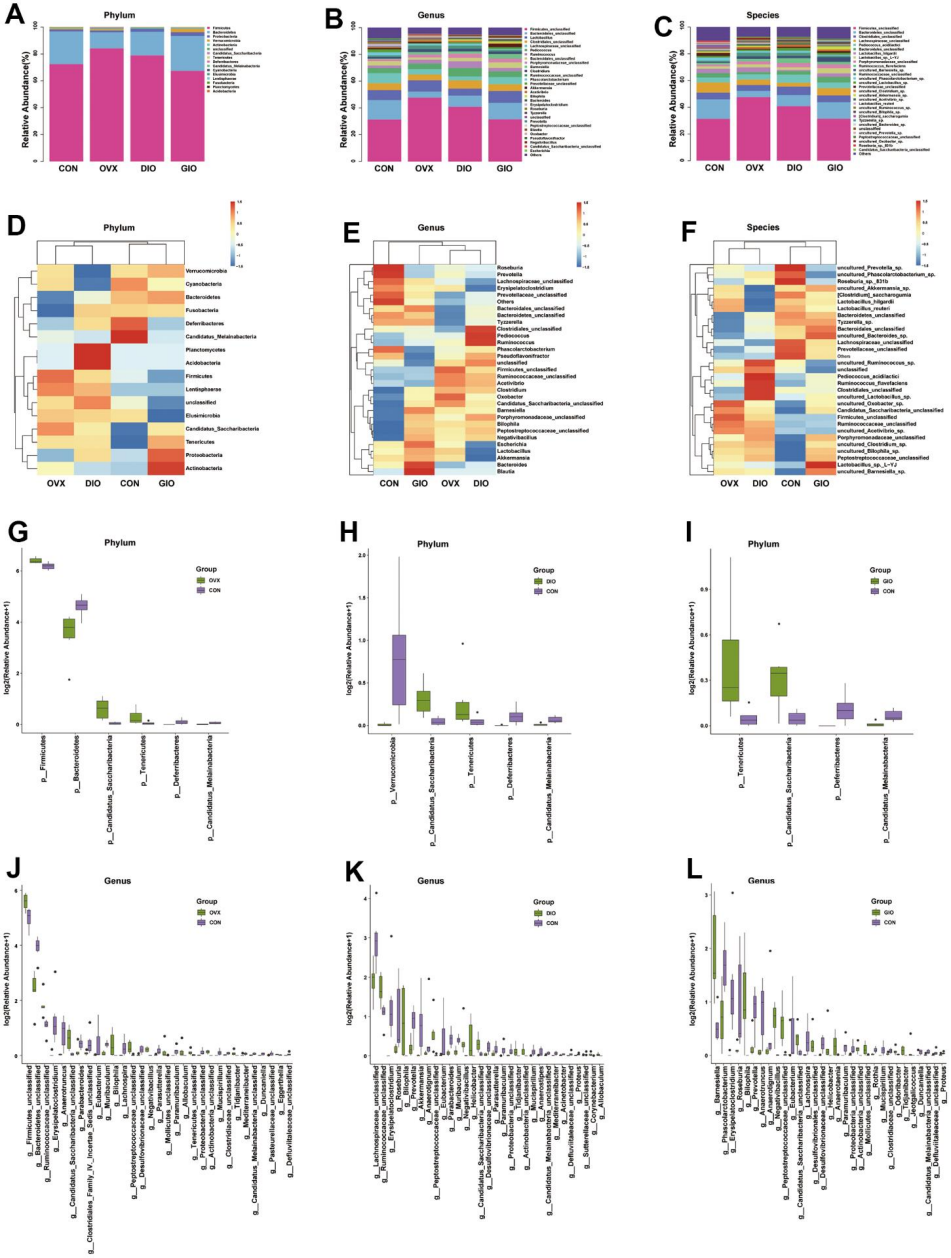
(**A**–**C**) Stacked bar chart showing bacterial phylum, genus, and species in GM. (**D**–**F**) Heat map showing bacterial phylum, genus, and species. (**G**–**I**) Significant phylum-level GM differences. (**J**–**L**) Significant genus-level GM differences; n = 6.

A Venn diagram visualized the common and unique amplicon sequence variants (ASVs) and the changes in the GM among the various treatment groups. The CON, OVX, DIO, and GIO groups had 2,449, 2,689, 2,683, and 1,176 unique ASVs while all four groups shared 553 ASVs (Figure 3A). We calculated the ASV abundances and plotted and compared the rarefaction curves for different samples to directly display bacterial species diversity and reflect the rationality of sequencing data. Flat rarefaction curves indicate reasonable sequencing data. An α-diversity analysis revealed that the rarefaction curves for the Shannon, Chao1, Goods_coverage, observed_species, and Simpson indices were all smooth (Figure 3B and Supplementary Figure 1A–1D). Beta-diversity analyses disclose and compare species diversity among various environmental communities. A principal coordinate analysis (PCoA) is a type of β-diversity analysis. Here, a PCoA was performed to demonstrate the differences among the four groups in terms of their gut microbiota. The samples with high similarity of community structure were clustered together by PCOA analysis, while the samples with great difference of community structure were far apart. The PCoA of the 2D and 3D models indicated that each OP group was induced by different factors distributed across various regions compared with the CON group. Although the samples from each OP group were assigned to approximately the same region, there were some differences between them. (Figure 3C, 3D). We then analyzed the GM community structure. We obtained the phylum-, class-, order-, family-, genus-, and species-level abundances and displayed them in the form of stacked bar charts and heat maps. The distributions of the various bacterial taxa of the OVX, DIO, and GIO groups differed from those of the CON group (Figure 4A–4F and Supplementary Figure 2A–2F). We also investigated the significant bacterial phylum- to species-level differences among the OVX, DIO, GIO, and CON groups (Figure 4G–4L and Supplementary Figure 2G–2O). At the phylum level, p__Candidatus_Saccharibacteria, p__Firmicutes, and p__Tenericutes were more abundant while p__Deferribacteres, p__Candidatus_Melainabacteria, and p__Bacteroidetes were less abundant in the OVX group compared with the CON group. Relative changes in p__Firmicutes and p__Bacteroidetes abundance were characteristic of the OVX group (Supplementary Table 1). However, p__Candidatus_Saccharibacteria and p__Tenericutes were more abundant while p__Candidatus_Melainabacteria, p__Verrucomicrobia, and p__Deferribacteres were less abundant in the DIO group compared with the CON group. Relative change in p__Verrucomicrobia abundance was characteristic of the DIO group (Supplementary Table 1). Moreover, p__Tenericutes and p__Candidatus_Saccharibacteria were more abundant while p__Candidatus_Melainabacteria and p__Deferribacteres were less abundant in the GIO group compared with the CON group (Supplementary Table 1). There were 36, 34, and 36 differential bacterial genera in the OVX, DIO, and GIO groups compared to the CON group (Supplementary Table 2). The taxonomic cladogram generated by linear discriminant analysis (LDA) effect size (LEfSe) visualized the relative differences among groups in terms of bacterial species abundance (Supplementary Figure 3A–3F).

### Differentially abundant metabolites among the various OP models in rats

**Figure 5:**
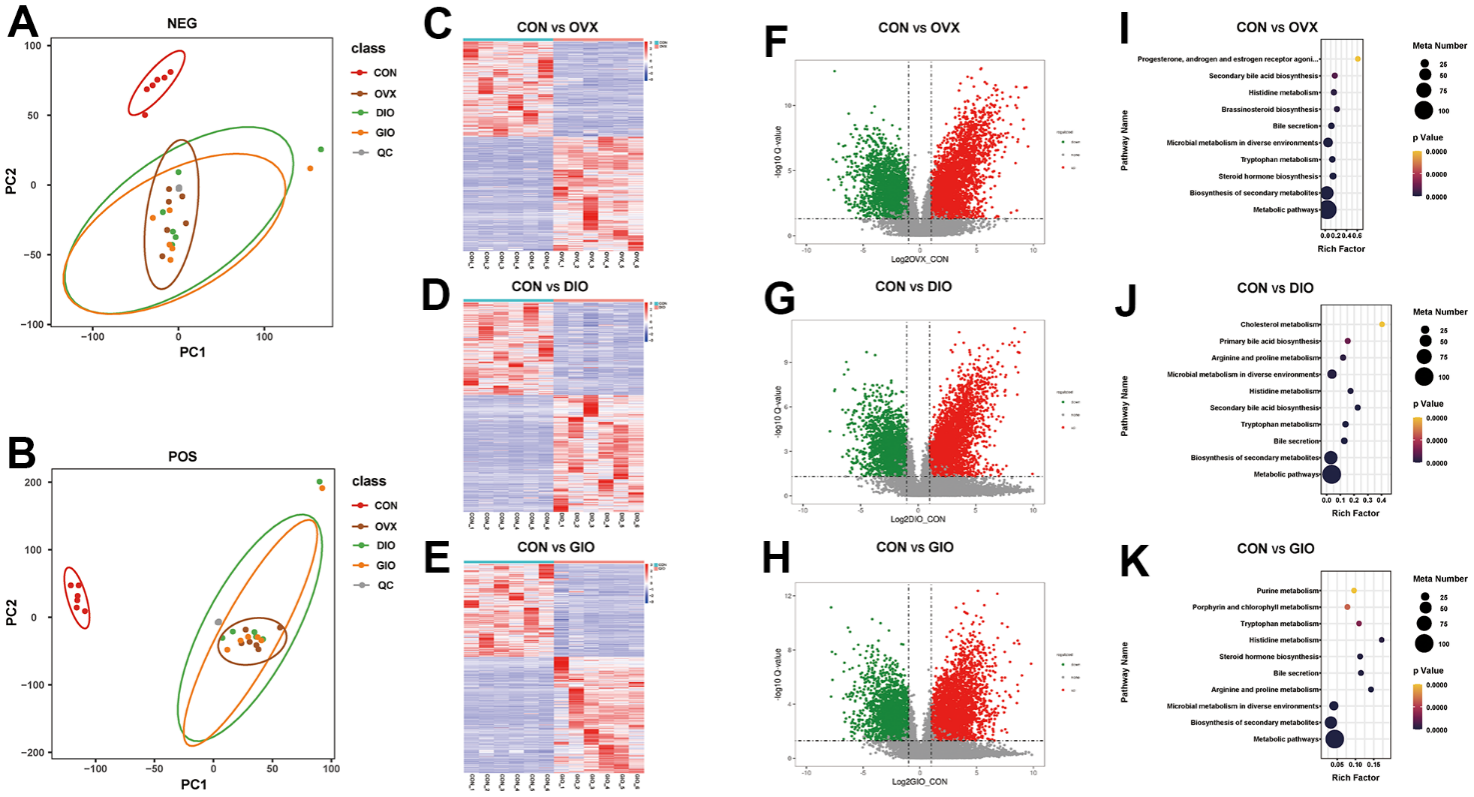
(**A**, **B**) PCA of fecal metabolites. (**C**–**E**) Heat map of differential fecal metabolites. (**F**–**H**) Volcano map of differential fecal metabolites. (**I**–**K**) Bubble diagram of KEGG enrichment analysis; n = 6.

A principal component analysis (PCA) is a commonly used type of multivariate analysis that identifies potential metabolomic markers within a large amount of data. Each point on a PCA graph represents a sample, and the similarity among samples decreases with increasing distance between points on the plot. Point clustering and separation indicate that the observed variables have high and low degrees of similarity, respectively. For the OVX, DIO, and GIO groups, the metabolites in positive and negative ion modes were in two distinct regions compared to the CON group. Thus, the metabolites were significantly altered in the OVX, DIO, and GIO groups relative to the CON group (Figure 5A, 5B). The heat maps and volcano maps generated the same results (Figure 5C–5H). In positive ion mode, and compared to the CON group, there were (a) 4,942 differential metabolites of which 2,755 were upregulated and 2,178 were downregulated in the OVX group; (b) 4,579 differential metabolites of which 2,620 were upregulated and 1,959 were downregulated in the DIO group; and (c) 5,041 differential metabolites of which 2,857 were upregulated and 2,178 were downregulated in the GIO group. In negative ion mode, and compared to the CON group, there were (a) 2,190 differential metabolites of which 1,134 were upregulated and 1,056 were downregulated in the OVX group; (b) 2,110 differential metabolites of which 1,111 were upregulated and 999 were downregulated in the DIO group; and (c) 2,398 differential metabolites of which 1,278 were upregulated and 1,120 were downregulated in the GIO group. A KEGG enrichment analysis was then performed on the differential metabolites and the top ten metabolic pathways and metabolites in the various OP models in rats (Figure 5I–5K and Supplementary Tables 3–8). Both lipid and amino acid metabolism may play important roles in osteoporosis progression.

### Correlation analyses of differential genus-level gut microbiota abundance and fecal metabolomes associated with lipid and amino acid metabolism

**Figure 6:**
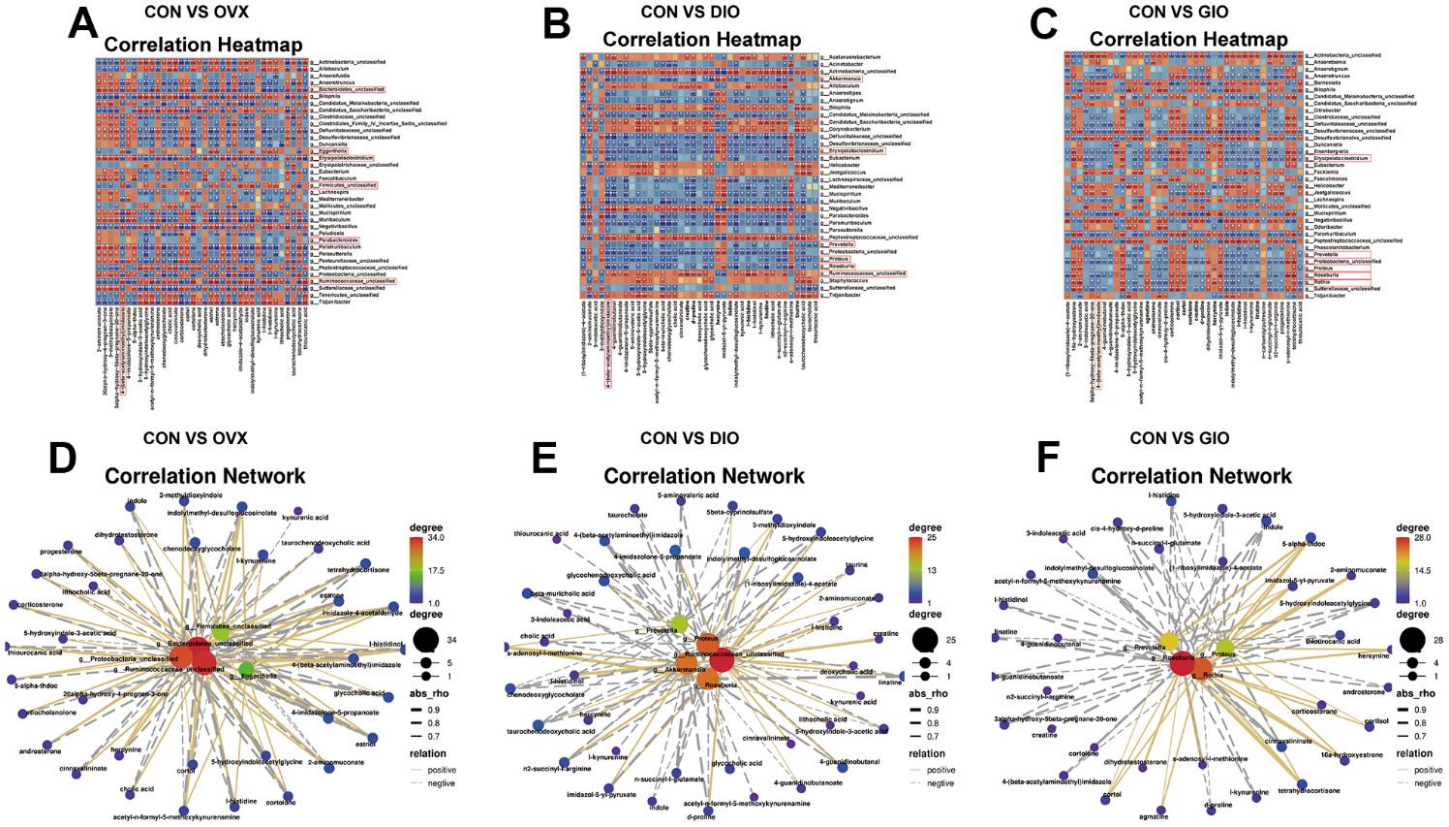
(**A**–**C**) Correlation heat map between differential bacterial genera and fecal metabolites associated with lipid and amino acid metabolism in top ten KEGG enrichment analysis; |r| > 0.6; P < 0.05. (**D**–**F**) Correlation network map of differential bone metabolism-related bacterial genera and fecal metabolites associated with lipid and amino acid metabolism in top ten KEGG enrichment analysis; |r| > 0.6; P < 0.05; n = 6.

To investigate the microbiota-metabolite interactions associated with each type of OP, we evaluated the correlations among differential bacterial genera and the top ten fecal metabolites related to lipid and amino acid metabolism according to the KEGG enrichment analysis. We plotted a correlation heat map (Figure 6A–6C; (|r| > 0.6, P < 0.05). Compared with the CON group, the differential bacterial genera included (a) g__Bacteroidetes_unclassified, g__Ruminococcaceae_unclassified, g__Parabacteroides, g__Firmicutes_unclassified, and g__Eggerthella in the OVX group, (b) g__Proteus, g__Akkermansia, g__Ruminococcaceae_unclassified, g__Roseburia, and g__Prevotella in the DIO group, and (c) g__Rothia, g__Roseburia, g__Proteus, and g__Prevotella in the GIO group. The foregoing taxa were closely related to bone metabolism. Changes in g__Bacteroidetes_unclassified, g__Firmicutes_unclassified, and g__Eggerthella were exclusive to the OVX group, a change in g__Akkermansia was specific to the DIO group, and a change in g__Rothia was unique to the GIO group. We then constructed a correlation network to disclose the major interactions among the differential bacterial genera associated with bone metabolism and the differential metabolites related to lipid and amino acid metabolism (Figure 6D–6F). The correlation heat map and network map revealed that the differential metabolite 4-(β-acetylaminoethyl) imidazole was negatively correlated with g__Bacteroidetes_unclassified (r = -0.783; P = 0.004), g__Akkermansia (r = -0.832; P = 0.001), and g__Rothia (r = -0.636; P = 0.030) but positively correlated with g__Firmicutes_unclassified (r = 0.727; P = 0.01) and g__Eggerthella (r = 0.748; P = 0.007). Hence, 4-(β-acetylaminoethyl) imidazole may be a principal metabolite associated with OP.

## DISCUSSION

The GM plays vital roles in maintaining human health [21]. Disorders of the GM may cause various chronic conditions including obesity, metabolic dysfunction, neuropathies, malnutrition, cancers, and cardiovascular diseases (CVD) [30]. Evidence from clinical and animal studies indicates that changes in the composition of the GM and its metabolites are closely associated with OP [23, 31–41]. Based on the interaction between the GM and OP, it was proposed that the former is a potential therapeutic target of the latter [42]. However, there are several different types of OP, and the composition of the GM and the metabolites characteristic of each form of OP are unknown. In the present study, we constructed three animal models to simulate postmenopausal, disuse-induced, and glucocorticoid-induced OP. We integrated 16S rDNA sequencing and untargeted metabolomics to explore the GM composition and metabolites characteristic of each type of OP.

Bacterial high-throughput sequencing based on 16S rDNA is used to study the microbial community composition, diversity, abundance, and structure in an environmental sample. It also analyzes the relationship between microorganisms and the environment or host in which they reside. Traditional microbial research relies on laboratory culture. However, not all environmental or symbiotic microorganisms can be propagated or studied in this way. In contrast, 16S amplicon and other high-throughput sequencing can investigate these bacteria and their interactions with their hosts or ambient environment. Our 16S rDNA sequencing and β-diversity analyses revealed that the GM differed among the three types of OP models in rats. Our subsequent species and significant difference analyses confirmed that the observed changes in the GM composition were different to each type of OP. There were five genera related to bone metabolism between the OVX and CON groups, and the changes in g__Bacteroidetes_unclassified, g__Firmicutes_unclassified, and g__Eggerthella were unique in the OVX. Firmicutes and Bacteroidetes are the major phyla in the GM and comprise 80% of the total microbiome [43]. The animal OVX model was characterized by relatively higher Firmicutes and lower Bacteroidetes abundance than other OP models [39, 40]. These findings were consistent with the changes detected in the present study. The foregoing alterations may serve as biomarkers of postmenopausal OP [40]. *Eggerthella* is a component of normal human microflora. Imbalances in its abundance are related to various diseases [44]. *Eggerthella* abundance is comparatively higher in patients with OP [45]. Members of Family *Eggerthellaceae* played vital roles in a mouse postmenopausal OP model [39]. *Eggerthella* can activate Th17 lymphocytes in the gut [46]. These cells are the main effectors of OP pathogenesis. They secrete interleukin (IL)-17 which, in turn, induces the NF-κB ligand/receptor activator of NF-κB/osteoprotegerin (RANKL/RANK/OPG) system and, by extension, promotes osteoclastogenesis and bone resorption [47, 48]. Therefore, *Eggerthella* may play an important role in postmenopausal OP. There were five genera related to bone metabolism between the DIO and CON groups, and the observed changes in g__Akkermansia abundance were unique in the DIO. In the gut microbiota, *Akkermansia* (Phylum Verrucomicrobia) influences the onset and progression of several diseases [49] and maintains intestinal barrier homeostasis [50]. The loss of *Akkermansia* impairs intestinal integrity, increases intestinal leakage, and, by extension, promotes metabolic endotoxemia, inflammation, and insulin resistance [51]. *Akkermansia* abundance was reduced in patients with osteoporosis and osteopenia [52, 53]. Here, *Akkermansia* abundance was comparatively lower in the DIO model. *Akkermansia* is osteoprotective, is positively correlated with bone mass, and could serve as a probiotic for OP prevention or treatment [54]. Our findings suggest that a decrease in the abundance of *Akkermansia* might play a key role in the progression of disuse OP. There were four genera related to bone metabolism between the GIO and CON groups, and the changes in g__Rothia abundance were unique in the GIO. Abnormal *Rothia* abundance may be associated with various infectious and alcoholic liver diseases [55, 56]. *Rothia* abundance is positively correlated with an anti-OP effect. It transforms xylose, galactose, raffinose, and glucose into the short-chain fatty acid (SCFA) butyric acid [57]. In general, SCFAs participate in bone metabolism regulation. Butyric acid inhibits and promotes osteoclast and osteoblast differentiation, respectively, from bone marrow mesenchymal stem cells (BMSCs) [58, 59]. Here, we observed a relative decrease in *Rothia* abundance in the GIO group which may be associated with the pathogenesis of GIO.

Liquid chromatography-mass spectrometry (LC-MS)-based metabolomics is used to identify and quantify the small molecules produced by normal microbial metabolism and elucidate their functions [60]. The GM secretes metabolites that link it to the skeletal system and regulate distant organs [61]. In the present study, we used untargeted LC-MS metabolomics to detect the fecal metabolites in the various OP models in rats. We discovered that among the top ten metabolic pathways and metabolites, lipid and amino acid metabolism play important roles in OP progression. Osteoporosis and osteopenia often co-exist with disorders of lipid metabolism [62]. When adipocyte numbers and volumes increase in the bone marrow, alterations to the microenvironment within the bone marrow cavity may perturb lipid metabolism there, inhibit BMSC osteoblastogenesis, promote BMSC osteoclastogenesis, and eventually lead to OP [63]. Hence, the improvement of lipid metabolism promotes osteoblastogenesis while inhibiting osteoclastogenesis [64]. Here, the lipid metabolism disorders associated with different types of OP models in rats involved mainly the biosynthesis of primary and secondary bile acids and steroid hormones. Bile acids have been associated with OP [37, 39]. There might be a correlation between OP and the circulating amino acids that play important roles in bone metabolism [65]. Abnormal amino acid metabolism may promote the occurrence and development of OP, and patients with OP often exhibit it [66]. The GM may regulate OP-related amino acid metabolism and could, therefore, serve as a target for OP intervention [67]. Here, the various OP models in rats were characterized by aberrant histidine, tryptophan, arginine, and proline metabolism. Intestinal bacteria decarboxylate the basic amino acid histidine to histamine which plays important roles in immunoregulation [68]. Histamine activates histamine H1 type receptor (H1R) which suppresses osteoblastogenesis and mineralization [69]. Histidine metabolism is at least partially implicated in bone formation [65]. In the present study, all three OP models in rats presented abnormal histidine metabolism. Correlation heat maps and networks among the differential bacterial genera and metabolites in all three OP models in rats showed that the histidine metabolism intermediate 4-(β-acetylaminoethyl) imidazole was closely associated with the bacterial genera related to bone metabolism. Thus, the bone loss induced by different types of OP models in rats might be connected to an increase in 4-(β-acetylaminoethyl) imidazole.

The present study had several limitations. Firstly, the sample size was relatively small. Hence, the results of this work provide only a few references to explore and compare intestinal microecology in different forms of OP. For this reason, future investigations must validate the findings of the work herein by using larger sample sizes. Moreover, future research should endeavor to elucidate the causal and regulatory relationships among the GM, its metabolites, and the various types of OP.

## CONCLUSIONS

The present work empirically demonstrated that each type of OP is closely, characteristically, and uniquely related to the GM and its metabolites. To the best of our knowledge, the present study is the first to characterize the GM and the changes in their metabolites associated with different types of OP models in rats. The results of this investigation may provide novel insights into the effects of the GM on the onset and progression of OP. Future research should aim to validate the findings made herein and determine how they may be applied toward safe and efficacious clinical therapies against different forms of OP.

## Supplementary Material

Supplementary Figures

Supplementary Tables

## References

[1] Ayub N, Faraj M, Ghatan S, Reijers JA, Napoli N, Oei L. The Treatment Gap in Osteoporosis. J Clin Med. 2021; 10:3002. 10.3390/jcm10133002 34279485 PMC8268346

[2] Pisani P, Renna MD, Conversano F, Casciaro E, Di Paola M, Quarta E, Muratore M, Casciaro S. Major osteoporotic fragility fractures: Risk factor updates and societal impact. World J Orthop. 2016; 7:171–81. 10.5312/wjo.v7.i3.171 27004165 PMC4794536

[3] Jain S, Camacho P. Use of bone turnover markers in the management of osteoporosis. Curr Opin Endocrinol Diabetes Obes. 2018; 25:366–72. 10.1097/MED.0000000000000446 30299435

[4] Akkawi I, Zmerly H. Osteoporosis: Current Concepts. Joints. 2018; 6:122–7. 10.1055/s-0038-1660790 30051110 PMC6059859

[5] Noh JY, Yang Y, Jung H. Molecular Mechanisms and Emerging Therapeutics for Osteoporosis. Int J Mol Sci. 2020; 21:7623. 10.3390/ijms21207623 33076329 PMC7589419

[6] Salari N, Ghasemi H, Mohammadi L, Behzadi MH, Rabieenia E, Shohaimi S, Mohammadi M. The global prevalence of osteoporosis in the world: a comprehensive systematic review and meta-analysis. J Orthop Surg Res. 2021; 16:609. 10.1186/s13018-021-02772-0 34657598 PMC8522202

[7] Komori T. Animal models for osteoporosis. Eur J Pharmacol. 2015; 759:287–94. 10.1016/j.ejphar.2015.03.028 25814262

[8] Ou L, Kang W, Zhang J, Liang Z, Li M, Gao F, Chen L. Effects of Rehmannia glutinosa polysaccharides on bone tissue structure and skeletal muscle atrophy in rats with disuse. Acta Cir Bras. 2021; 36:e360403. 10.1590/ACB360403 34008744 PMC8128353

[9] Shane E, Burr D, Abrahamsen B, Adler RA, Brown TD, Cheung AM, Cosman F, Curtis JR, Dell R, Dempster DW, Ebeling PR, Einhorn TA, Genant HK, et al. Atypical subtrochanteric and diaphyseal femoral fractures: second report of a task force of the American Society for Bone and Mineral Research. J Bone Miner Res. 2014; 29:1–23. 10.1002/jbmr.1998 23712442

[10] Khan AA, Morrison A, Hanley DA, Felsenberg D, McCauley LK, O’Ryan F, Reid IR, Ruggiero SL, Taguchi A, Tetradis S, Watts NB, Brandi ML, Peters E, et al, and International Task Force on Osteonecrosis of the Jaw. Diagnosis and management of osteonecrosis of the jaw: a systematic review and international consensus. J Bone Miner Res. 2015; 30:3–23. 10.1002/jbmr.2405 25414052

[11] Cresci GA, Bawden E. Gut Microbiome: What We Do and Don’t Know. Nutr Clin Pract. 2015; 30:734–46. 10.1177/0884533615609899 26449893 PMC4838018

[12] Behera J, Ison J, Tyagi SC, Tyagi N. The role of gut microbiota in bone homeostasis. Bone. 2020; 135:115317. 10.1016/j.bone.2020.115317 32169602 PMC8457311

[13] Dominguez-Bello MG, Godoy-Vitorino F, Knight R, Blaser MJ. Role of the microbiome in human development. Gut. 2019; 68:1108–14. 10.1136/gutjnl-2018-317503 30670574 PMC6580755

[14] Ni J, Wu GD, Albenberg L, Tomov VT. Gut microbiota and IBD: causation or correlation? Nat Rev Gastroenterol Hepatol. 2017; 14:573–84. 10.1038/nrgastro.2017.88 28743984 PMC5880536

[15] Hand TW, Vujkovic-Cvijin I, Ridaura VK, Belkaid Y. Linking the Microbiota, Chronic Disease, and the Immune System. Trends Endocrinol Metab. 2016; 27:831–43. 10.1016/j.tem.2016.08.003 27623245 PMC5116263

[16] Van de Wiele T, Van Praet JT, Marzorati M, Drennan MB, Elewaut D. How the microbiota shapes rheumatic diseases. Nat Rev Rheumatol. 2016; 12:398–411. 10.1038/nrrheum.2016.85 27305853

[17] Ohlsson C, Sjögren K. Effects of the gut microbiota on bone mass. Trends Endocrinol Metab. 2015; 26:69–74. 10.1016/j.tem.2014.11.004 25497348

[18] Locantore P, Del Gatto V, Gelli S, Paragliola RM, Pontecorvi A. The Interplay between Immune System and Microbiota in Osteoporosis. Mediators Inflamm. 2020; 2020:3686749. 10.1155/2020/3686749 32184701 PMC7061131

[19] Rescigno M. Intestinal microbiota and its effects on the immune system. Cell Microbiol. 2014; 16:1004–13. 10.1111/cmi.12301 24720613

[20] Nakashima T, Hayashi M, Fukunaga T, Kurata K, Oh-Hora M, Feng JQ, Bonewald LF, Kodama T, Wutz A, Wagner EF, Penninger JM, Takayanagi H. Evidence for osteocyte regulation of bone homeostasis through RANKL expression. Nat Med. 2011; 17:1231–4. 10.1038/nm.2452 21909105

[21] Hao ML, Wang GY, Zuo XQ, Qu CJ, Yao BC, Wang DL. Gut microbiota: an overlooked factor that plays a significant role in osteoporosis. J Int Med Res. 2019; 47:4095–103. 10.1177/0300060519860027 31436117 PMC6753565

[22] Li J, Ho WT, Liu C, Chow SK, Ip M, Yu J, Wong HS, Cheung WH, Sung JJ, Wong RM. The role of gut microbiota in bone homeostasis. Bone Joint Res. 2021; 10:51–9. 10.1302/2046-3758.101.BJR-2020-0273.R1 33448869 PMC7845471

[23] Sun Y, Zhang HJ, Chen R, Zhao HB, Lee WH. 16S rDNA analysis of the intestinal microbes in osteoporotic rats. Biosci Microbiota Food Health. 2021; 40:156–67. 10.12938/bmfh.2020-065 34285861 PMC8279887

[24] Monzem S, Javaheri B, de Souza RL, Pitsillides AA. Sciatic neurectomy-related cortical bone loss exhibits delayed onset yet stabilises more rapidly than trabecular bone. Bone Rep. 2021; 15:101116. 10.1016/j.bonr.2021.101116 34471655 PMC8387754

[25] Li J, Yang M, Lu C, Han J, Tang S, Zhou J, Li Y, Ming T, Wang ZJ, Su X. Tuna Bone Powder Alleviates Glucocorticoid-Induced Osteoporosis via Coregulation of the NF-κB and Wnt/β-Catenin Signaling Pathways and Modulation of Gut Microbiota Composition and Metabolism. Mol Nutr Food Res. 2020; 64:e1900861. 10.1002/mnfr.201900861 31953894

[26] Qiao X, Zhang K, Li X, Lv Z, Wei W, Zhou R, Yan L, Pan Y, Yang S, Sun X, Li P, Xu C, Feng Y, Tian Z. Gut microbiota and fecal metabolic signatures in rat models of disuse-induced osteoporosis. Front Cell Infect Microbiol. 2022; 12:1018897. 10.3389/fcimb.2022.1018897 36590590 PMC9798431

[27] Kanehisa M, Goto S. KEGG: kyoto encyclopedia of genes and genomes. Nucleic Acids Res. 2000; 28:27–30. 10.1093/nar/28.1.27 10592173 PMC102409

[28] Kanehisa M. Toward understanding the origin and evolution of cellular organisms. Protein Sci. 2019; 28:1947–51. 10.1002/pro.3715 31441146 PMC6798127

[29] Kanehisa M, Furumichi M, Sato Y, Kawashima M, Ishiguro-Watanabe M. KEGG for taxonomy-based analysis of pathways and genomes. Nucleic Acids Res. 2023; 51:D587–92. 10.1093/nar/gkac963 36300620 PMC9825424

[30] Song S, Guo Y, Yang Y, Fu D. Advances in pathogenesis and therapeutic strategies for osteoporosis. Pharmacol Ther. 2022; 237:108168. 10.1016/j.pharmthera.2022.108168 35283172

[31] Ni JJ, Yang XL, Zhang H, Xu Q, Wei XT, Feng GJ, Zhao M, Pei YF, Zhang L. Assessing causal relationship from gut microbiota to heel bone mineral density. Bone. 2021; 143:115652. 10.1016/j.bone.2020.115652 32971307

[32] Cheng S, Qi X, Ma M, Zhang L, Cheng B, Liang C, Liu L, Li P, Kafle OP, Wen Y, Zhang F. Assessing the Relationship Between Gut Microbiota and Bone Mineral Density. Front Genet. 2020; 11:6. 10.3389/fgene.2020.00006 32082367 PMC7005253

[33] Zhou T, Wang M, Ma H, Li X, Heianza Y, Qi L. Dietary Fiber, Genetic Variations of Gut Microbiota-derived Short-chain Fatty Acids, and Bone Health in UK Biobank. J Clin Endocrinol Metab. 2021; 106:201–10. 10.1210/clinem/dgaa740 33051670 PMC8186524

[34] Ozaki D, Kubota R, Maeno T, Abdelhakim M, Hitosugi N. Association between gut microbiota, bone metabolism, and fracture risk in postmenopausal Japanese women. Osteoporos Int. 2021; 32:145–56. 10.1007/s00198-020-05728-y 33241467 PMC7755620

[35] He J, Xu S, Zhang B, Xiao C, Chen Z, Si F, Fu J, Lin X, Zheng G, Yu G, Chen J. Gut microbiota and metabolite alterations associated with reduced bone mineral density or bone metabolic indexes in postmenopausal osteoporosis. Aging (Albany NY). 2020; 12:8583–604. 10.18632/aging.103168 32392181 PMC7244073

[36] Huang R, Liu P, Bai Y, Huang J, Pan R, Li H, Su Y, Zhou Q, Ma R, Zong S, Zeng G. Changes in the gut microbiota of osteoporosis patients based on 16S rRNA gene sequencing: a systematic review and meta-analysis. J Zhejiang Univ Sci B. 2022; 23:1002–13. 10.1631/jzus.B2200344 36518053 PMC9758719

[37] Lu L, Tang M, Li J, Xie Y, Li Y, Xie J, Zhou L, Liu Y, Yu X. Gut Microbiota and Serum Metabolic Signatures of High-Fat-Induced Bone Loss in Mice. Front Cell Infect Microbiol. 2021; 11:788576. 10.3389/fcimb.2021.788576 35004355 PMC8727351

[38] Cheng M, Tan B, Wu X, Liao F, Wang F, Huang Z. Gut Microbiota Is Involved in Alcohol-Induced Osteoporosis in Young and Old Rats Through Immune Regulation. Front Cell Infect Microbiol. 2021; 11:636231. 10.3389/fcimb.2021.636231 34336709 PMC8317599

[39] Wen K, Tao L, Tao Z, Meng Y, Zhou S, Chen J, Yang K, Da W, Zhu Y. Fecal and Serum Metabolomic Signatures and Microbial Community Profiling of Postmenopausal Osteoporosis Mice Model. Front Cell Infect Microbiol. 2020; 10:535310. 10.3389/fcimb.2020.535310 33330117 PMC7728697

[40] Ma S, Qin J, Hao Y, Shi Y, Fu L. Structural and functional changes of gut microbiota in ovariectomized rats and their correlations with altered bone mass. Aging (Albany NY). 2020; 12:10736–53. 10.18632/aging.103290 32484785 PMC7346027

[41] Ma S, Qin J, Hao Y, Fu L. Association of gut microbiota composition and function with an aged rat model of senile osteoporosis using 16S rRNA and metagenomic sequencing analysis. Aging (Albany NY). 2020; 12:10795–808. 10.18632/aging.103293 32487781 PMC7346068

[42] Tu Y, Yang R, Xu X, Zhou X. The microbiota-gut-bone axis and bone health. J Leukoc Biol. 2021; 110:525–37. 10.1002/JLB.3MR0321-755R 33884666

[43] Lay C, Sutren M, Rochet V, Saunier K, Doré J, Rigottier-Gois L. Design and validation of 16S rRNA probes to enumerate members of the Clostridium leptum subgroup in human faecal microbiota. Environ Microbiol. 2005; 7:933–46. 10.1111/j.1462-2920.2005.00763.x 15946290

[44] Jiang S, E J, Wang D, Zou Y, Liu X, Xiao H, Wen Y, Chen Z. Eggerthella lenta bacteremia successfully treated with ceftizoxime: case report and review of the literature. Eur J Med Res. 2021; 26:111. 10.1186/s40001-021-00582-y 34544476 PMC8454090

[45] Wei M, Li C, Dai Y, Zhou H, Cui Y, Zeng Y, Huang Q, Wang Q. High-Throughput Absolute Quantification Sequencing Revealed Osteoporosis-Related Gut Microbiota Alterations in Han Chinese Elderly. Front Cell Infect Microbiol. 2021; 11:630372. 10.3389/fcimb.2021.630372 33996619 PMC8120270

[46] Alexander M, Ang QY, Nayak RR, Bustion AE, Sandy M, Zhang B, Upadhyay V, Pollard KS, Lynch SV, Turnbaugh PJ. Human gut bacterial metabolism drives Th17 activation and colitis. Cell Host Microbe. 2022; 30:17–30.e9. 10.1016/j.chom.2021.11.001 34822777 PMC8785648

[47] Wu D, Cline-Smith A, Shashkova E, Perla A, Katyal A, Aurora R. T-Cell Mediated Inflammation in Postmenopausal Osteoporosis. Front Immunol. 2021; 12:687551. 10.3389/fimmu.2021.687551 34276675 PMC8278518

[48] Ono T, Hayashi M, Sasaki F, Nakashima T. RANKL biology: bone metabolism, the immune system, and beyond. Inflamm Regen. 2020; 40:2. 10.1186/s41232-019-0111-3 32047573 PMC7006158

[49] Xue C, Li G, Gu X, Su Y, Zheng Q, Yuan X, Bao Z, Lu J, Li L. Health and Disease: *Akkermansia muciniphila*, the Shining Star of the Gut Flora. Research (Wash D C). 2023; 6:0107. 10.34133/research.0107 37040299 PMC10079265

[50] Zhou JC, Zhang XW. Akkermansia muciniphila: a promising target for the therapy of metabolic syndrome and related diseases. Chin J Nat Med. 2019; 17:835–41. 10.1016/S1875-5364(19)30101-3 31831130

[51] Bodogai M, O’Connell J, Kim K, Kim Y, Moritoh K, Chen C, Gusev F, Vaughan K, Shulzhenko N, Mattison JA, Lee-Chang C, Chen W, Carlson O, et al. Commensal bacteria contribute to insulin resistance in aging by activating innate B1a cells. Sci Transl Med. 2018; 10:eaat4271. 10.1126/scitranslmed.aat4271 30429354 PMC6445267

[52] Keshavarz Azizi Raftar S, Hoseini Tavassol Z, Amiri M, Ejtahed HS, Zangeneh M, Sadeghi S, Ashrafian F, Kariman A, Khatami S, Siadat SD. Assessment of fecal *Akkermansia muciniphila* in patients with osteoporosis and osteopenia: a pilot study. J Diabetes Metab Disord. 2021; 20:279–84. 10.1007/s40200-021-00742-1 34222066 PMC8212221

[53] Qin Q, Yan S, Yang Y, Chen J, Yan H, Li T, Gao X, Wang Y, Li A, Wang S, Ding S. The Relationship Between Osteoporosis and Intestinal Microbes in the Henan Province of China. Front Cell Dev Biol. 2021; 9:752990. 10.3389/fcell.2021.752990 34869341 PMC8638085

[54] Liu JH, Chen CY, Liu ZZ, Luo ZW, Rao SS, Jin L, Wan TF, Yue T, Tan YJ, Yin H, Yang F, Huang FY, Guo J, et al. Extracellular Vesicles from Child Gut Microbiota Enter into Bone to Preserve Bone Mass and Strength. Adv Sci (Weinh). 2021; 8:2004831. 10.1002/advs.202004831 33977075 PMC8097336

[55] Mahobia N, Chaudhary P, Kamat Y. Rothia prosthetic knee joint infection: report and mini-review. New Microbes New Infect. 2013; 1:2–5. 10.1002/2052-2975.7 25356316 PMC4184483

[56] Plaza-Díaz J, Solís-Urra P, Rodríguez-Rodríguez F, Olivares-Arancibia J, Navarro-Oliveros M, Abadía-Molina F, Álvarez-Mercado AI. The Gut Barrier, Intestinal Microbiota, and Liver Disease: Molecular Mechanisms and Strategies to Manage. Int J Mol Sci. 2020; 21:8351. 10.3390/ijms21218351 33171747 PMC7664383

[57] Yin Y, Zhu F, Pan M, Bao J, Liu Q, Tao Y. A Multi-Omics Analysis Reveals Anti-Osteoporosis Mechanism of Four Components from Crude and Salt-Processed *Achyranthes bidentata* Blume in Ovariectomized Rats. Molecules. 2022; 27:5012. 10.3390/molecules27155012 35956964 PMC9370352

[58] Tyagi AM, Yu M, Darby TM, Vaccaro C, Li JY, Owens JA, Hsu E, Adams J, Weitzmann MN, Jones RM, Pacifici R. The Microbial Metabolite Butyrate Stimulates Bone Formation via T Regulatory Cell-Mediated Regulation of WNT10B Expression. Immunity. 2018; 49:1116–31.e7. 10.1016/j.immuni.2018.10.013 30446387 PMC6345170

[59] Jafarnejad S, Djafarian K, Fazeli MR, Yekaninejad MS, Rostamian A, Keshavarz SA. Effects of a Multispecies Probiotic Supplement on Bone Health in Osteopenic Postmenopausal Women: A Randomized, Double-blind, Controlled Trial. J Am Coll Nutr. 2017; 36:497–506. 10.1080/07315724.2017.1318724 28628374

[60] Bauermeister A, Mannochio-Russo H, Costa-Lotufo LV, Jarmusch AK, Dorrestein PC. Mass spectrometry-based metabolomics in microbiome investigations. Nat Rev Microbiol. 2022; 20:143–60. 10.1038/s41579-021-00621-9 34552265 PMC9578303

[61] Zaiss MM, Jones RM, Schett G, Pacifici R. The gut-bone axis: how bacterial metabolites bridge the distance. J Clin Invest. 2019; 129:3018–28. 10.1172/JCI128521 31305265 PMC6668676

[62] Tian L, Yu X. Lipid metabolism disorders and bone dysfunction--interrelated and mutually regulated (review). Mol Med Rep. 2015; 12:783–94. 10.3892/mmr.2015.3472 25760577 PMC4438959

[63] He H, Zhang Y, Sun Y, Zhang Y, Xu J, Yang Y, Chen J. Folic Acid Attenuates High-Fat Diet-Induced Osteoporosis Through the AMPK Signaling Pathway. Front Cell Dev Biol. 2022; 9:791880. 10.3389/fcell.2021.791880 35047504 PMC8762056

[64] Kushwaha P, Wolfgang MJ, Riddle RC. Fatty acid metabolism by the osteoblast. Bone. 2018; 115:8–14. 10.1016/j.bone.2017.08.024 28863948 PMC5832496

[65] Lu L, Chen X, Liu Y, Yu X. Gut microbiota and bone metabolism. FASEB J. 2021; 35:e21740. 10.1096/fj.202100451R 34143911

[66] Lee WC, Guntur AR, Long F, Rosen CJ. Energy Metabolism of the Osteoblast: Implications for Osteoporosis. Endocr Rev. 2017; 38:255–66. 10.1210/er.2017-00064 28472361 PMC5460680

[67] Ling CW, Miao Z, Xiao ML, Zhou H, Jiang Z, Fu Y, Xiong F, Zuo LS, Liu YP, Wu YY, Jing LP, Dong HL, Chen GD, et al. The Association of Gut Microbiota With Osteoporosis Is Mediated by Amino Acid Metabolism: Multiomics in a Large Cohort. J Clin Endocrinol Metab. 2021; 106:e3852–64. 10.1210/clinem/dgab492 34214160

[68] Barcik W, Wawrzyniak M, Akdis CA, O’Mahony L. Immune regulation by histamine and histamine-secreting bacteria. Curr Opin Immunol. 2017; 48:108–13. 10.1016/j.coi.2017.08.011 28923468

[69] Sun Y, Peng X, Li Y, Ma H, Li D, Shi X. The effects of histamine H1 type receptor (H1R) in regulating osteoblastic cell differentiation and mineralization. Artif Cells Nanomed Biotechnol. 2019; 47:1281–7. 10.1080/21691401.2019.1596924 30942635

