# Supplementary material for: Gut microbial community and fecal metabolomic signatures in different types of osteoporosis animal models
Source: Aging (Albany NY). 2024 Jan 26;16(2):1192–217. doi: 10.18632/aging.205396 (PMC10866450; doi:10.18632/aging.205396)
Supplement: Supplementary Figures [file aging-16-205396-s001.pdf]

SUPPLEMENTARY FIGURES

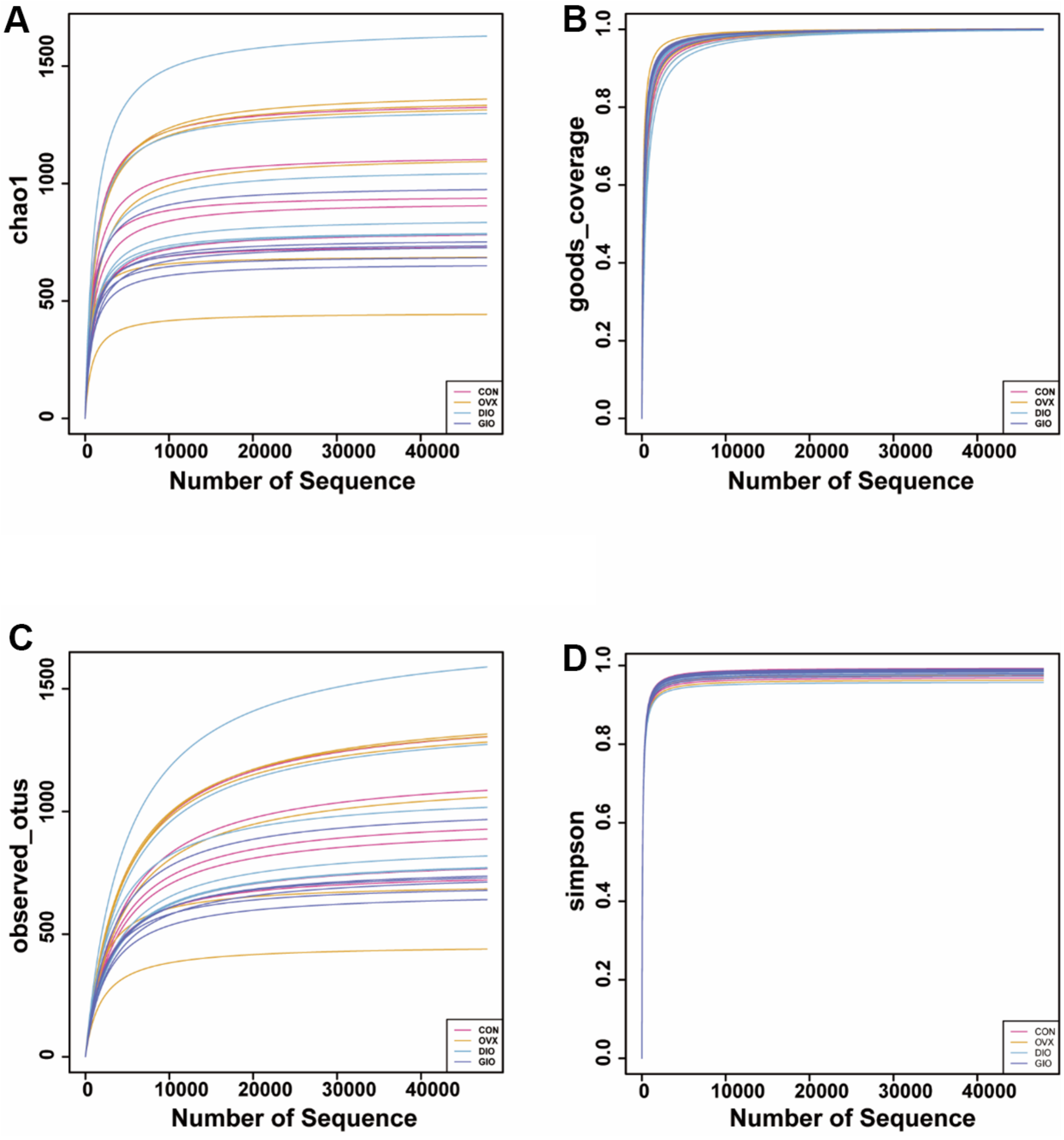

Supplementary Figure 1. Rarefaction curves of alpha diversity analysis among four groups. (**A**) Rarefaction curves in chao1. (**B**) Rarefaction curves in Goods_coverage. (**C**) Rarefaction curves in observed_species. (**D**) Rarefaction curves in simpson. n = 6.

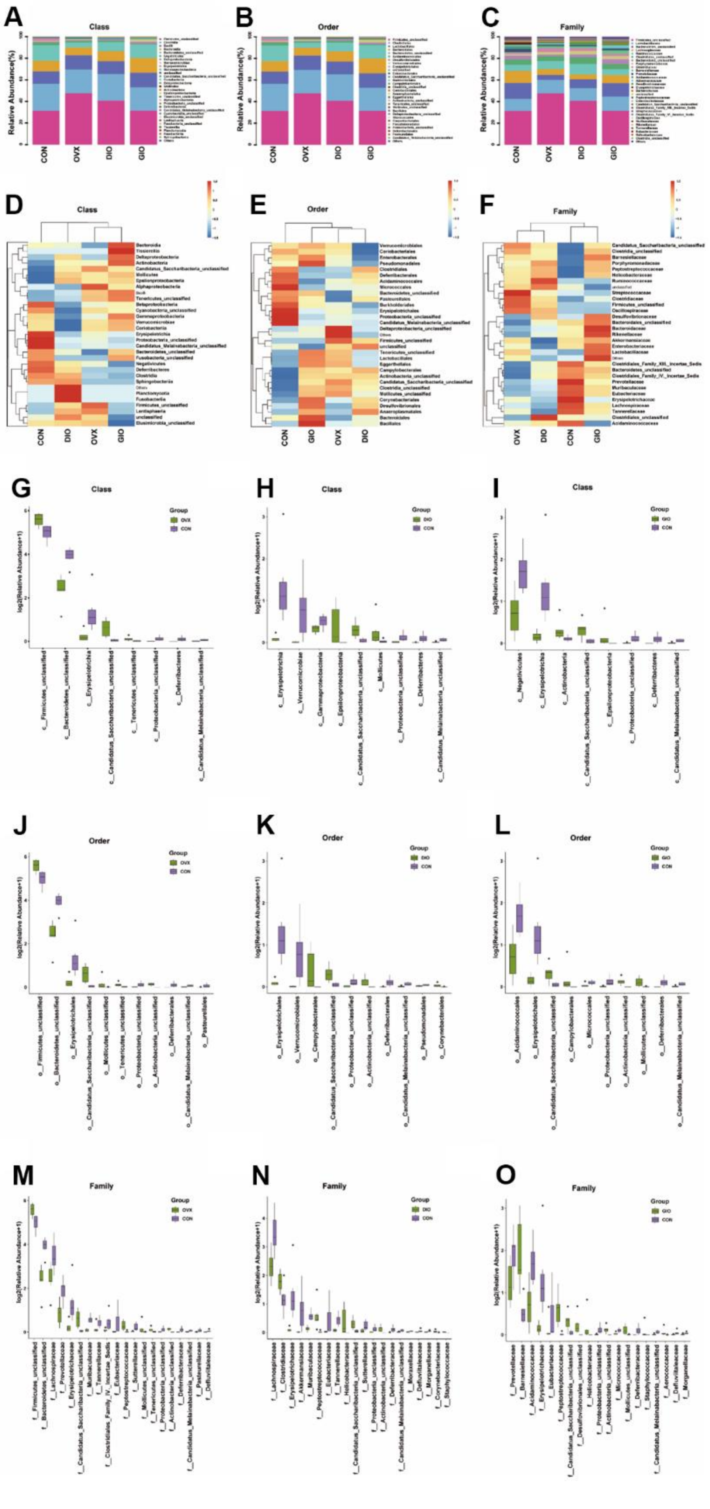

Supplementary Figure 2. The stacked bar charts, heat maps and significant differences in GM of other levels among the four groups. (**A**–**C**) class, order, family-level stacked bar charts. (**D**–**F**) class, order, family-level heat maps. (**G**–**I**) class-level significant different GM. (**J**–**L**) order-level significant different GM. (**M**–**O**) family-level significant different GM. n = 6.

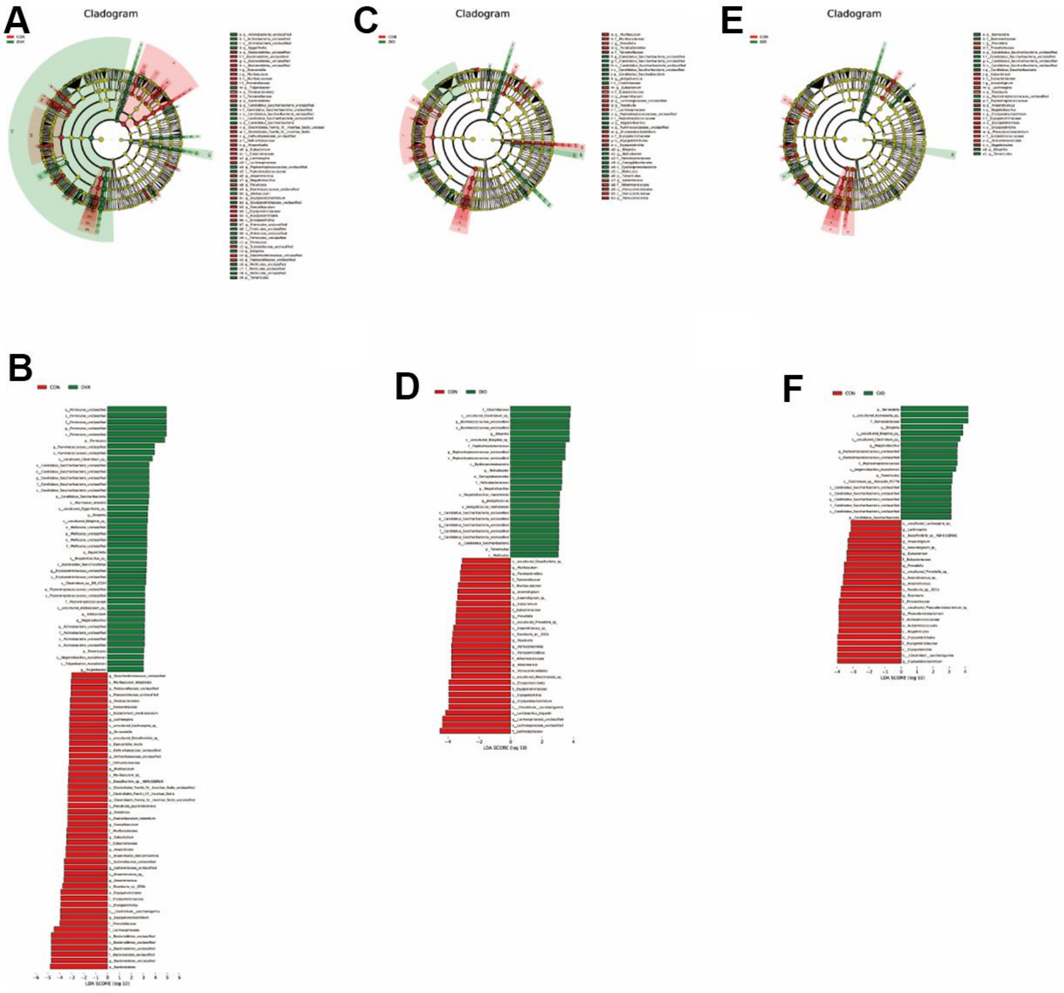

Supplementary Figure 3. LEfSe analysis of gut microbiota in the CON, OVX, DIO and GIO groups. (**A**, **B**) CON vs OVX. (**C**, **D**) CON vs DIO. (**E**, **F**) CON vs GIO. Red represents increased microbiota; green represents decreased microbiota. LDA score > 4.0, n = 6.
