# Supplementary material for: Gut microbial community and fecal metabolomic signatures in different types of osteoporosis animal models
Source: Aging (Albany NY). 2024 Jan 26;16(2):1192–217. doi: 10.18632/aging.205396 (PMC10866450; doi:10.18632/aging.205396)
Supplement: Supplementary Tables [file aging-16-205396-s002.pdf]

SUPPLEMENTARY TABLES

Supplementary Table 1. Significant difference of GM at the phylum level.

CON VS	Phylum	log2FC	p_value	Significance	Regulation
OVX	p__Candidatus_Saccharibacteria	4.11	0.01	yes	up
OVX	p__Deferribacteres	-Inf	0.01	yes	down
OVX	p__Candidatus_Melainabacteria	-2.40	0.01	yes	down
OVX	p__Firmicutes	0.22	0.02	yes	up
OVX	p__Bacteroidetes	-1.02	0.02	yes	down
OVX	p__Tenericutes	2.75	0.04	yes	up
DIO	p__Candidatus_Melainabacteria	-2.70	0.01	yes	down
DIO	p__Candidatus_Saccharibacteria	2.87	0.01	yes	up
DIO	p__Verrucomicrobia	-6.97	0.01	yes	down
DIO	p__Deferribacteres	-6.38	0.01	yes	down
DIO	p__Tenericutes	2.72	0.04	yes	up
GIO	p__Candidatus_Melainabacteria	-2.63	0.01	yes	down
GIO	p__Deferribacteres	-Inf	0.01	yes	down
GIO	p__Tenericutes	3.37	0.02	yes	up
GIO	p__Candidatus_Saccharibacteria	2.95	0.03	yes	up

Supplementary Table 2. Significant difference of GM at the genus level.

CON VS	Genus	log2FC	p_value	Significance	Regulation
OVX	g__Bilophila	Inf	0.00	yes	up
OVX	g__Actinobacteria_unclassified	Inf	0.00	yes	up
OVX	g__Tidjanibacter	Inf	0.00	yes	up
OVX	g__Paramuribaculum	-Inf	0.00	yes	down
OVX	g__Erysipelatoclostridium	-6.22	0.00	yes	down
OVX	g__Sutterellaceae_unclassified	-4.42	0.00	yes	down
OVX	g__Negativibacillus	5.41	0.00	yes	up
OVX	g__Bacteroidetes_unclassified	-1.65	0.00	yes	down
OVX	g__Ruminococcaceae_unclassified	1.28	0.00	yes	up
OVX	g__Candidatus_Saccharibacteria_unclassified	4.11	0.00	yes	up
OVX	g__Anaerotruncus	-4.98	0.00	yes	down
OVX	g__Muribaculum	-2.60	0.00	yes	down
OVX	g__Proteobacteria_unclassified	-3.26	0.00	yes	down
OVX	g__Pasteurellaceae_unclassified	-4.75	0.00	yes	down
OVX	g__Peptostreptococcaceae_unclassified	4.63	0.01	yes	up
OVX	g__Desulfovibrionaceae_unclassified	-3.84	0.01	yes	down
OVX	g__Defluviitaleaceae_unclassified	-3.22	0.01	yes	down
OVX	g__Clostridiaceae_unclassified	-1.86	0.01	yes	down
OVX	g__Parasutterella	-3.14	0.01	yes	down
OVX	g__Allobaculum	Inf	0.01	yes	up
OVX	g__Mucispirillum	-Inf	0.01	yes	down
OVX	g__Eubacterium	-6.67	0.01	yes	down
OVX	g__Candidatus_Melainabacteria_unclassified	-2.40	0.01	yes	down
OVX	g__Tenericutes_unclassified	2.67	0.01	yes	up
OVX	g__Lachnospira	-5.15	0.02	yes	down
OVX	g__Paludicola	-2.69	0.02	yes	down
OVX	g__Firmicutes_unclassified	0.60	0.02	yes	up
OVX	g__Clostridiales_Family_IV._Incertae_Sedis_unclassified	-2.76	0.02	yes	down
OVX	g__Faecalibaculum	-Inf	0.02	yes	down
OVX	g__Erysipelotrichaceae_unclassified	2.50	0.03	yes	up
OVX	g__Mollicutes_unclassified	5.86	0.03	yes	up
OVX	g__Duncaniella	-3.76	0.03	yes	down
OVX	g__Parabacteroides	-1.54	0.04	yes	down
OVX	g__Eggerthella	1.18	0.04	yes	up
OVX	g__Mediterraneibacter	-2.04	0.05	yes	down
OVX	g__Anaerofustis	-3.64	0.05	yes	down
DIO	g__Erysipelatoclostridium	-Inf	0.00	yes	down
DIO	g__Actinobacteria_unclassified	Inf	0.00	yes	up
DIO	g__Paramuribaculum	-7.79	0.00	yes	down
DIO	g__Peptostreptococcaceae_unclassified	5.86	0.00	yes	up
DIO	g__Proteobacteria_unclassified	-3.60	0.00	yes	down
DIO	g__Negativibacillus	6.59	0.00	yes	up
DIO	g__Defluviitaleaceae_unclassified	-3.26	0.00	yes	down
DIO	g__Parabacteroides	-2.78	0.00	yes	down
DIO	g__Muribaculum	-2.10	0.00	yes	down
DIO	g__Candidatus_Melainabacteria_unclassified	-2.70	0.01	yes	down
DIO	g__Proteus	-1.70	0.01	yes	down
DIO	g__Candidatus_Saccharibacteria_unclassified	2.87	0.01	yes	up
DIO	g__Bilophila	Inf	0.01	yes	up
DIO	g__Helicobacter	Inf	0.01	yes	up
DIO	g__Tidjanibacter	Inf	0.01	yes	up

DIO	g__Akkermansia	-7.09	0.01	yes	down
DIO	g__Ruminococcaceae_unclassified	1.06	0.01	yes	up
DIO	g__Roseburia	-3.52	0.01	yes	down
DIO	g__Corynebacterium	5.19	0.01	yes	up
DIO	g__Mucispirillum	-6.38	0.01	yes	down
DIO	g__Eubacterium	-5.57	0.02	yes	down
DIO	g__Desulfovibrionaceae_unclassified	-2.64	0.02	yes	down
DIO	g__Lachnospiraceae_unclassified	-1.37	0.02	yes	down
DIO	g__Prevotella	-2.36	0.02	yes	down
DIO	g__Anaerotignum	-3.67	0.02	yes	down
DIO	g__Sutterellaceae_unclassified	-2.98	0.02	yes	down
DIO	g__Anaerostipes	-5.14	0.02	yes	down
DIO	g__Allobaculum	Inf	0.02	yes	up
DIO	g__Jeotgalicoccus	Inf	0.02	yes	up
DIO	g__Staphylococcus	Inf	0.02	yes	up
DIO	g__Acetanaerobacterium	1.81	0.03	yes	up
DIO	g__Mediterraneibacter	-2.96	0.04	yes	down
DIO	g__Parasutterella	-1.90	0.04	yes	down
DIO	g__Acinetobacter	-1.14	0.04	yes	down
GIO	g__Desulfovibrionales_unclassified	Inf	0.00	yes	up
GIO	g__Actinobacteria_unclassified	Inf	0.00	yes	up
GIO	g__Paramuribaculum	-Inf	0.00	yes	down
GIO	g__Sutterellaceae_unclassified	-Inf	0.00	yes	down
GIO	g__Erysipelatoclostridium	-7.45	0.00	yes	down
GIO	g__Desulfovibrionaceae_unclassified	-7.35	0.00	yes	down
GIO	g__Peptostreptococcaceae_unclassified	5.89	0.00	yes	up
GIO	g__Negativibacillus	7.30	0.00	yes	up
GIO	g__Proteobacteria_unclassified	-4.01	0.00	yes	down
GIO	g__Eisenbergiella	-4.95	0.00	yes	down
GIO	g__Candidatus_Melainabacteria_unclassified	-2.63	0.01	yes	down
GIO	g__Barnesiella	2.81	0.01	yes	up
GIO	g__Roseburia	-3.99	0.01	yes	down
GIO	g__Rothia	-2.37	0.01	yes	down
GIO	g__Bilophila	Inf	0.01	yes	up
GIO	g__Helicobacter	Inf	0.01	yes	up
GIO	g__Mucispirillum	-Inf	0.01	yes	down
GIO	g__Defluviitaleaceae_unclassified	-3.22	0.01	yes	down
GIO	g__Phascolarctobacterium	-1.75	0.01	yes	down
GIO	g__Anaerotruncus	-4.50	0.01	yes	down
GIO	g__Lachnospira	-5.51	0.02	yes	down
GIO	g__Proteus	-1.82	0.02	yes	down
GIO	g__Prevotella	-2.63	0.02	yes	down
GIO	g__Clostridiaceae_unclassified	-1.62	0.02	yes	down
GIO	g__Mollicutes_unclassified	5.23	0.02	yes	up
GIO	g__Tidjanibacter	Inf	0.02	yes	up
GIO	g__Jeotgalicoccus	Inf	0.02	yes	up
GIO	g__Facklamia	Inf	0.02	yes	up
GIO	g__Faecalimonas	-Inf	0.02	yes	down
GIO	g__Citrobacter	-Inf	0.02	yes	down
GIO	g__Candidatus_Saccharibacteria_unclassified	2.95	0.03	yes	up
GIO	g__Eubacterium	-2.15	0.03	yes	down
GIO	g__Anaerotignum	-3.45	0.04	yes	down
GIO	g__Odoribacter	2.25	0.04	yes	up
GIO	g__Duncaniella	-1.85	0.05	yes	down
GIO	g__Anaerotaenia	6.77	0.05	yes	up

Supplementary Table 3. Details of TOP10 KEGG pathways enrichment analysis in OVX vs CON.

Level 2	Level 1	Pathway	KEGG	P-value
Lipid metabolism	Metabolism	Steroid hormone biosynthesis	map00140	3.60E-11
Amino acid metabolism	Metabolism	Tryptophan metabolism	map00380	1.64E-08
Amino acid metabolism	Metabolism	Histidine metabolism	map00340	5.61E-07
Lipid metabolism	Metabolism	Secondary bile acid biosynthesis	map00121	1.87E-06

Supplementary Table 4. Details of TOP10 KEGG pathways enrichment analysis in DIO vs CON.

Level 2	Level 1	Pathway	KEGG	P-value
Amino acid metabolism	Metabolism	Tryptophan metabolism	map00380	1.09E-09
Lipid metabolism	Metabolism	Secondary bile acid biosynthesis	map00121	3.04E-09
Amino acid metabolism	Metabolism	Histidine metabolism	map00340	2.86E-08
Amino acid metabolism	Metabolism	Arginine and proline metabolism	map00330	1.27E-07
Lipid metabolism	Metabolism	Primary bile acid biosynthesis	map00120	5.61E-07

Supplementary Table 5. Details of TOP10 KEGG pathways enrichment analysis in DIO vs CON.

Level 2	Level 1	Pathway	KEGG	P-value
Amino acid metabolism	Metabolism	Arginine and proline metabolism	map00330	2.56E-09
Lipid metabolism	Metabolism	Steroid hormone biosynthesis	map00140	3.32E-08
Amino acid metabolism	Metabolism	Histidine metabolism	map00340	8.81E-08
Amino acid metabolism	Metabolism	Tryptophan metabolism	map00380	7.45E-07

Supplementary Table 6. Differential metabolites in lipid and amino acid metabolism related pathways of OVX vs CON.

ID	Metabolite	KEGG	Level 1	Level 2	Pathway	Regulated
pos-3.491_264.11055	3alpha-hydroxy-5beta-pregnane-20-one	C05480	Metabolism	Lipid metabolism	Steroid hormone biosynthesis	down
pos-3.427_310.1525	estriol	C05141	Metabolism	Lipid metabolism	Steroid hormone biosynthesis	up
pos-4.685_316.23923	20alpha-hydroxy-4-pregnen-3-one	C04042	Metabolism	Lipid metabolism	Steroid hormone biosynthesis	down
pos-4.685_290.22366	dihydrotestosterone	C03917	Metabolism	Lipid metabolism	Steroid hormone biosynthesis	down
pos-4.192_366.24003	cortolone	C05481	Metabolism	Lipid metabolism	Steroid hormone biosynthesis	up
pos-4.685_334.24965	5-alpha-thdoc	C13713	Metabolism	Lipid metabolism	Steroid hormone biosynthesis	down
neg-5.169_368.25556	cortol	C05482	Metabolism	Lipid metabolism	Steroid hormone biosynthesis	down
pos-7.166_346.21112	corticosterone	C02140	Metabolism	Lipid metabolism	Steroid hormone biosynthesis	down
neg-3.348_270.15783	estrone	C00468	Metabolism	Lipid metabolism	Steroid hormone biosynthesis	up
pos-4.213_364.22429	tetrahydrocortisone	C05470	Metabolism	Lipid metabolism	Steroid hormone biosynthesis	down
pos-5.793_314.22367	progesterone	C00410	Metabolism	Lipid metabolism	Steroid hormone biosynthesis	down
pos-4.61_272.2134	androsterone	C00523	Metabolism	Lipid metabolism	Steroid hormone biosynthesis	down
pos-4.684_272.2134	etiocholanolone	C04373	Metabolism	Lipid metabolism	Steroid hormone biosynthesis	down
neg-3.181_157.03705	2-aminomuconate	C02220	Metabolism	Amino acid metabolism	Tryptophan metabolism	down
neg-3.496_189.04223	kynurenic acid	C01717	Metabolism	Amino acid metabolism	Tryptophan metabolism	down
pos-3.177_208.08446	l-kynurenine	C00328	Metabolism	Amino acid metabolism	Tryptophan metabolism	up
pos-4.768_117.05781	indole	C00463	Metabolism	Amino acid metabolism	Tryptophan metabolism	up
neg-4.778_368.1038	indolylmethyl-desulfoglucosinolate	C16517	Metabolism	Amino acid metabolism	Tryptophan metabolism	up
neg-3.873_248.0793	5-hydroxyindoleacetylglycine	C05832	Metabolism	Amino acid metabolism	Tryptophan metabolism	up
neg-4.661_300.03853	cinnavalininate	C05640	Metabolism	Amino acid metabolism	Tryptophan metabolism	up
pos-5.054_191.05781	5-hydroxyindole-3-acetic acid	C05635	Metabolism	Amino acid metabolism	Tryptophan metabolism	up

pos-0.922_163.06303	3-methyldioxyindole	C05834	Metabolism	Amino acid metabolism	Tryptophan metabolism	down
pos-3.491_264.11055	acetyl-n-formyl-5-methoxykynurenamine	C05642	Metabolism	Amino acid metabolism	Tryptophan metabolism	up
neg-4.245_198.12493	hercynine	C05575	Metabolism	Amino acid metabolism	Histidine metabolism	down
pos-3.76_155.06925	l-histidine	C00135	Metabolism	Amino acid metabolism	Histidine metabolism	up
pos-4.017_110.04778	imidazole-4-acetaldehyde	C05130	Metabolism	Amino acid metabolism	Histidine metabolism	up
neg-0.836_156.05273	4-imidazolone-5-propanoate	C03680	Metabolism	Amino acid metabolism	Histidine metabolism	down
pos-3.133_170.01483	thiourocanic acid	C05571	Metabolism	Amino acid metabolism	Histidine metabolism	up
pos-1.66_141.08994	l-histidinol	C00860	Metabolism	Amino acid metabolism	Histidine metabolism	up
pos-0.746_153.08976	4-(beta-acetylaminoethyl)imidazole	C05135	Metabolism	Amino acid metabolism	Histidine metabolism	up
neg-5.502_392.292	deoxycholic acid	C04483	Metabolism	Lipid metabolism	Secondary bile acid biosynthesis	up
pos-6.719_358.28622	lithocholic acid	C03990	Metabolism	Lipid metabolism	Secondary bile acid biosynthesis	up
neg-4.726_499.2962	taurochenodeoxycholic acid	C05465	Metabolism	Lipid metabolism	Secondary bile acid biosynthesis	up
neg-4.879_408.28703	cholic acid	C00695	Metabolism	Lipid metabolism	Secondary bile acid biosynthesis	up
pos-4.831_449.31236	chenodeoxyglycocholate	C05466	Metabolism	Lipid metabolism	Secondary bile acid biosynthesis	up
neg-4.322_465.3082	glycocholic acid	C01921	Metabolism	Lipid metabolism	Secondary bile acid biosynthesis	up

Supplementary Table 7. Differential metabolites in lipid and amino acid metabolism related pathways of DIO vs CON.

ID	Metabolite	KEGG	Level 1	Level 2	Pathway	Regulated
pos-3.491_264.11055	acetyl-n-formyl-5-methoxykynurenamine	C05642	Metabolism	Amino acid metabolism	Tryptophan metabolism	up
pos-0.922_163.06303	3-methyldioxyindole	C05834	Metabolism	Amino acid metabolism	Tryptophan metabolism	down
neg-3.181_157.03705	2-aminomuconate	C02220	Metabolism	Amino acid metabolism	Tryptophan metabolism	down
pos-3.177_208.08446	l-kynurenine	C00328	Metabolism	Amino acid metabolism	Tryptophan metabolism	up
neg-4.778_368.1038	indolylmethyl-desulfoglucosinolate	C16517	Metabolism	Amino acid metabolism	Tryptophan metabolism	up
pos-4.611_117.05776	indole	C00463	Metabolism	Amino acid metabolism	Tryptophan metabolism	up
pos-5.678_175.06289	3-indoleacetic acid	C00954	Metabolism	Amino acid metabolism	Tryptophan metabolism	up
neg-3.449_189.0422	kynurenic acid	C01717	Metabolism	Amino acid metabolism	Tryptophan metabolism	up
pos-5.054_191.05781	5-hydroxyindole-3-acetic acid	C05635	Metabolism	Amino acid metabolism	Tryptophan metabolism	up
neg-4.661_300.03853	cinnavalininate	C05640	Metabolism	Amino acid metabolism	Tryptophan metabolism	up
neg-3.873_248.0793	5-hydroxyindoleacetylglycine	C05832	Metabolism	Amino acid metabolism	Tryptophan metabolism	up
neg-4.712_449.31359	glycochenodeoxycholic acid	C05466	Metabolism	Lipid metabolism	Secondary bile acid biosynthesis	up
pos-4.831_449.31236	chenodeoxyglycocholate	C05466	Metabolism	Lipid metabolism	Secondary bile acid biosynthesis	up
neg-4.322_465.3082	glycocholic acid	C01921	Metabolism	Lipid metabolism	Secondary bile acid biosynthesis	up
neg-4.726_499.2962	taurochenodeoxycholic acid	C05465	Metabolism	Lipid metabolism	Secondary bile acid biosynthesis	up
pos-6.596_358.28617	lithocholic acid	C03990	Metabolism	Lipid metabolism	Secondary bile acid biosynthesis	up
neg-3.962_515.29062	taurocholate	C05122	Metabolism	Lipid metabolism	Secondary bile acid biosynthesis	up
neg-4.825_392.292	deoxycholic acid	C04483	Metabolism	Lipid metabolism	Secondary bile acid biosynthesis	up
neg-4.879_408.28703	cholic acid	C00695	Metabolism	Lipid metabolism	Secondary bile acid biosynthesis	up
neg-4.194_454.29268	beta-muricholic acid	C17726	Metabolism	Lipid metabolism	Secondary bile acid biosynthesis	up
neg-4.245_198.12493	hercynine	C05575	Metabolism	Amino acid metabolism	Histidine metabolism	down
pos-0.925_141.08985	l-histidinol	C00860	Metabolism	Amino acid metabolism	Histidine metabolism	up
neg-3.987_156.05322	4-imidazolone-5-propanoate	C03680	Metabolism	Amino acid metabolism	Histidine metabolism	up
pos-0.746_153.08976	4-(beta-acetylaminoethyl)imidazole	C05135	Metabolism	Amino acid metabolism	Histidine metabolism	up
pos-0.896_258.08461	(1-ribosylimidazole)-4-acetate	C05131	Metabolism	Amino acid metabolism	Histidine metabolism	up
pos-0.917_155.06892	l-histidine	C00135	Metabolism	Amino acid metabolism	Histidine metabolism	up
neg-1.357_154.03746	imidazol-5-yl-pyruvate	C03277	Metabolism	Amino acid metabolism	Histidine metabolism	down
pos-3.133_170.01483	thiourocanic acid	C05571	Metabolism	Amino acid metabolism	Histidine metabolism	up
pos-0.802_129.08996	4-guanidinobutanal	C02647	Metabolism	Amino acid metabolism	Arginine and proline metabolism	up
pos-0.926_115.06295	d-proline	C00763	Metabolism	Amino acid metabolism	Arginine and proline metabolism	up
pos-0.907_259.11617	linatine	C05939	Metabolism	Amino acid	Arginine and proline	up

				metabolism	metabolism	
pos-1.38_145.08487	4-guanidinobutanoate	C01035	Metabolism	Amino acid metabolism	Arginine and proline metabolism	up
neg-3.183_247.06878	n-succinyl-l-glutamate	C05931	Metabolism	Amino acid metabolism	Arginine and proline metabolism	up
neg-4.193_399.14628	s-adenosyl-l-methionine	C00019	Metabolism	Amino acid metabolism	Arginine and proline metabolism	down
pos-0.882_131.06919	creatine	C00300	Metabolism	Amino acid metabolism	Arginine and proline metabolism	up
pos-0.847_274.12698	n2-succinyl-l-arginine	C03296	Metabolism	Amino acid metabolism	Arginine and proline metabolism	up
pos-4.102_100.05215	5-aminovaleric acid	C00431	Metabolism	Amino acid metabolism	Arginine and proline metabolism	up
neg-0.807_125.01433	taurine	C00245	Metabolism	Lipid metabolism	Primary bile acid biosynthesis	up
neg-4.389_532.30619	5beta-cyprinolsulfate	C05468	Metabolism	Lipid metabolism	Primary bile acid biosynthesis	up

Supplementary Table 8. Differential metabolites in lipid and amino acid metabolism related pathways of GIO vs CON.

ID	Metabolite	KEGG	Level 1	Level 2	Pathway	Regulated
pos-0.907_259.11617	linatine	C05939	Metabolism	Amino acid metabolism	Arginine and proline metabolism	up
pos-3.536_145.08496	4-guanidinobutanoate	C01035	Metabolism	Amino acid metabolism	Arginine and proline metabolism	up
pos-0.926_115.06295	d-proline	C00763	Metabolism	Amino acid metabolism	Arginine and proline metabolism	up
neg-4.193_399.14628	s-adenosyl-l-methionine	C00019	Metabolism	Amino acid metabolism	Arginine and proline metabolism	down
neg-3.183_247.06878	n-succinyl-l-glutamate	C05931	Metabolism	Amino acid metabolism	Arginine and proline metabolism	up
pos-0.882_131.06919	creatine	C00300	Metabolism	Amino acid metabolism	Arginine and proline metabolism	up
pos-0.836_131.05794	cis-4-hydroxy-d-proline	C03440	Metabolism	Amino acid metabolism	Arginine and proline metabolism	up
pos-0.847_274.12698	n2-succinyl-l-arginine	C03296	Metabolism	Amino acid metabolism	Arginine and proline metabolism	up
pos-0.845_131.10558	n-carbamoylputrescine	C00436	Metabolism	Amino acid metabolism	Arginine and proline metabolism	down
pos-0.76_130.12149	agmatine	C00179	Metabolism	Amino acid metabolism	Arginine and proline metabolism	down
pos-0.802_129.08996	4-guanidinobutanal	C02647	Metabolism	Amino acid metabolism	Arginine and proline metabolism	up
pos-4.77_318.25491	3alpha-hydroxy-5beta-pregnane-20-one	C05480	Metabolism	Lipid metabolism	Steroid hormone biosynthesis	up
pos-4.192_366.24003	cortolone	C05481	Metabolism	Lipid metabolism	Steroid hormone biosynthesis	up
neg-4.004_368.25572	cortol	C05482	Metabolism	Lipid metabolism	Steroid hormone biosynthesis	down
pos-6.691_290.22366	dihydrotestosterone	C03917	Metabolism	Lipid metabolism	Steroid hormone biosynthesis	down
pos-4.213_364.22429	tetrahydrocortisone	C05470	Metabolism	Lipid metabolism	Steroid hormone biosynthesis	down
pos-6.495_362.2058	cortisol	C00735	Metabolism	Lipid metabolism	Steroid hormone biosynthesis	down

pos-7.166_346.21112	corticosterone	C02140	Metabolism	Lipid metabolism	Steroid hormone biosynthesis	down
pos-7.29_334.24973	5-alpha-thdoc	C13713	Metabolism	Lipid metabolism	Steroid hormone biosynthesis	down
pos-4.001_286.15639	16a-hydroxyestrone	C05300	Metabolism	Lipid metabolism	Steroid hormone biosynthesis	down
pos-4.61_314.22372	progesterone	C00410	Metabolism	Lipid metabolism	Steroid hormone biosynthesis	up
pos-4.219_272.21354	androsterone	C00523	Metabolism	Lipid metabolism	Steroid hormone biosynthesis	up
pos-3.133_170.01483	thiourocanic acid	C05571	Metabolism	Amino acid metabolism	Histidine metabolism	up
pos-3.76_155.06925	l-histidine	C00135	Metabolism	Amino acid metabolism	Histidine metabolism	up
neg-4.245_198.12493	hercynine	C05575	Metabolism	Amino acid metabolism	Histidine metabolism	down
neg-1.357_154.03746	imidazol-5-yl-pyruvate	C03277	Metabolism	Amino acid metabolism	Histidine metabolism	down
neg-0.836_156.05273	4-imidazolone-5-propanoate	C03680	Metabolism	Amino acid metabolism	Histidine metabolism	down
pos-0.896_258.08461	(1-ribosylimidazole)-4-acetate	C05131	Metabolism	Amino acid metabolism	Histidine metabolism	up
pos-0.746_153.08976	4-(beta-acetylaminoethyl)imidazole	C05135	Metabolism	Amino acid metabolism	Histidine metabolism	up
pos-0.925_141.08985	l-histidinol	C00860	Metabolism	Amino acid metabolism	Histidine metabolism	up
pos-3.177_208.08446	l-kynurenine	C00328	Metabolism	Amino acid metabolism	Tryptophan metabolism	up
pos-5.678_175.06289	3-indoleacetic acid	C00954	Metabolism	Amino acid metabolism	Tryptophan metabolism	up
neg-4.661_300.03853	cinnavalininate	C05640	Metabolism	Amino acid metabolism	Tryptophan metabolism	up
neg-4.778_368.1038	indolylmethyl-desulfoglucosinolate	C16517	Metabolism	Amino acid metabolism	Tryptophan metabolism	up
neg-3.873_248.0793	5-hydroxyindoleacetylglycine	C05832	Metabolism	Amino acid metabolism	Tryptophan metabolism	up
pos-4.768_117.05781	indole	C00463	Metabolism	Amino acid metabolism	Tryptophan metabolism	up
pos-5.054_191.05781	5-hydroxyindole-3-acetic acid	C05635	Metabolism	Amino acid metabolism	Tryptophan metabolism	up
pos-3.491_264.11055	acetyl-n-formyl-5-methoxykynurenamine	C05642	Metabolism	Amino acid metabolism	Tryptophan metabolism	up
neg-3.181_157.03705	2-aminomuconate	C02220	Metabolism	Amino acid metabolism	Tryptophan metabolism	down
